# Metabarcoding Reveals Fine Scale Patterns of Trophic Resource Use and Partitioning Along Gradients of Land Use and Deer Density in a Multi‐Species Ungulate Community

**DOI:** 10.1002/ece3.72365

**Published:** 2025-10-21

**Authors:** Robert Spitzer, Eric Coissac, Annika M. Felton, Marietjie Landman, Navinder J. Singh, Pierre Taberlet, Fredrik Widemo, Joris P. G. M. Cromsigt

**Affiliations:** ^1^ Department of Wildlife, Fish and Environmental Studies Swedish University of Agricultural Sciences Umeå Sweden; ^2^ Université Grenoble‐Alpes Université Savoie Mont Blanc, CNRS, LECA Grenoble France; ^3^ Southern Swedish Forest Research Centre, Faculty of Forest Sciences Swedish University of Agricultural Sciences Lomma Sweden; ^4^ Centre for African Conservation Ecology, Department of Zoology Nelson Mandela University Gqeberha South Africa; ^5^ The Arctic University Museum of Norway UiT—The Arctic University of Norway Tromsø Norway; ^6^ SLU Forest Damage Centre Swedish University of Agricultural Sciences Umeå Sweden

**Keywords:** cervid diets, dietary niche width, dietary overlap, DNA metabarcoding, trophic resource partitioning

## Abstract

Across the northern hemisphere, ungulates are expanding in range and abundance, forming novel communities in increasingly human‐modified landscapes. These shifts drive new interactions over available food resources, but patterns of resource use and partitioning in Europe's multi‐species systems remain poorly understood. This study examined seasonal diets and resource partitioning in diverse cervid communities (moose, roe deer, red deer, and fallow deer) across two Swedish landscapes (coastal‐boreal and boreo‐nemoral) differing in deer density and land use. Based on their foraging strategies, we expected (Hypothesis 1) diet richness and dietary niche width to be greater in intermediate feeders (red and fallow deer) than in browsers (moose and roe deer), (Hypothesis 2) trophic partitioning between browsers and intermediate feeders to be driven mainly by graminoid use, and (Hypothesis 3) intra‐ and interspecific overlap to vary with season, deer density, habitat diversity, and proportion of arable land. DNA metabarcoding of 2568 fecal samples showed that deer consumed plants from over 70 families, though diets were typically dominated by fewer than 10. *Vaccinium* shrubs were key forages year‐round, while birch and willow dominated during the growing season. Moose consumed large amounts of pine in spring and winter (> 50% in the boreo‐nemoral, 35%–40% in the coastal‐boreal landscape), with less during summer‐autumn (~15%). Forbs were important for smaller deer, especially in spring and summer‐autumn, and more heavily used in winter in the boreo‐nemoral landscape, likely due to supplementary feeding with human‐provided food like hay or silage. Spruce use was low overall (< 5%), with fallow deer showing the highest intake. Consistent with Hypothesis 1, diet richness and niche width increased from moose to fallow deer. In partial support of Hypothesis 2, principal coordinates analysis (PCoA) revealed that graminoids contributed to trophic partitioning, but the pattern was not a strict browser–intermediate feeder divide. Moose consistently separated from the smaller deer due to avoidance of graminoids and reliance on pine and juniper, while roe deer, although a browser, sometimes overlapped with red and fallow deer through greater use of graminoids. During winter in the coastal‐boreal landscape, wavy hairgrass ( *Avenella flexuosa* ) contributed to the significant separation between browsing roe deer and intermediate‐feeding red deer diets, consistent with Hypothesis 2. Diet overlap among smaller deer varied with season and landscape. Intraspecific overlap was the highest in moose and the lowest in fallow deer, declining during summer–autumn across species. Overlap was influenced by deer density, habitat diversity, and arable land, consistent with Hypothesis 3, but effects were species‐specific and explained only limited variation. Our results highlight the dietary plasticity of red and fallow deer, which may intensify resource competition with moose and roe deer in multi‐species systems, particularly where supplementary feeding is common. These insights support adaptive, multi‐species management of deer in northern ecosystems.

## Graphical abstract

We used DNA metabarcoding of 2,568 fecal samples to examine seasonal diets and resource partitioning among four deer species (moose, roe deer, red deer, fallow deer) across two Swedish landscapes. Deer consumed a wide range of plants, but diets were typically dominated by a few key taxa, with distinct dietary separation between moose and smaller deer. Results reveal species‐specific plasticity and seasonal shifts in resource use, emphasizing the need for adaptive, multi‐species management in changing northern ecosystems.

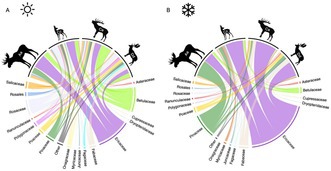

## 1 Introduction

Across the northern hemisphere, ungulate species are expanding their distributional ranges due to warmer winters, increased protection, and human‐altered landscapes that increase access to food resources, particularly through crops in agricultural lands and forest regenerations (Apollonio et al. 2010) and, historically, the eradication of large predators (Blossey and Hare 2022). In Sweden, for example, large‐scale rotational forestry and Scots pine ( *Pinus sylvestris* ) plantations have substantially increased the availability of this important winter browse for moose, contributing to strong population growth (Edenius et al. 2002). Decreased winter severity, with reductions in snow, substantially increases the suitability of winter habitat, facilitating range expansion at northern limits and higher elevations. For example, red deer ( *Cervus elaphus* ) show large expansions of suitable winter ranges under even moderate warming (Rivrud et al. 2019), while ibex ( *Capra ibex* ), chamois ( *Rupicapra rupicapra* ), and red deer in the Swiss Alps have shifted to higher elevations during snow‐free autumns (Büntgen et al. 2017). Similarly, the distribution of white‐tailed deer (*Odocoileus virginianus*) in boreal forests contracts during severe winters but rebounds under moderate conditions, with further expansion expected as winters become less severe (Fisher et al. 2020). In Alaska, moose range expansion has likewise been linked to warming and increased shrub habitat (Tape et al. 2016). In many places, such processes have led to the establishment of novel multi‐species ungulate communities, usually dominated by different deer species. These novel communities also lead to novel interactions over available food sources among the ungulate species in these communities. For example, in Europe, red deer and fallow deer ( *Dama dama* ) now occur as far north as 64 degrees latitude, which is much further north than their historical ranges. The establishment of both red and fallow deer this far north in Sweden was partly assisted by humans through escapes from enclosures during the 1980s and the subsequent establishment of expanding populations (Wengberg 2021). At this high latitude, both species engage in novel interactions with moose ( *Alces alces* ) and roe deer ( *Capreolus capreolus* ).

In a world of finite resources, ecologists have long been interested in the mechanisms that allow species to coexist. Interactions over available resources have classically been viewed as a major driver behind the structuring of large herbivore communities and can range from facilitation (Arsenault and Owen‐Smith 2002; Gordon 1988) to competitive interactions (Forsyth and Hickling 1998). Such interactions operate at multiple scales (e.g., spatial and temporal) and at different levels (e.g., intra‐ and interspecific), driving the processes of resource partitioning and niche separation. For example, spatial segregation in large herbivores has been observed where smaller species such as red hartebeest ( *Alcelaphus buselaphus* ) show stronger habitat selection than larger eland (*Tragelaphus oryx*) (Fortin et al. 2024), while temporal differentiation can occur through contrasting use of foraging areas, e.g., between zebra ( *Equus quagga* ) and wildebeest ( *Connochaetes taurinus* ) (Owen‐Smith et al. 2015). Interspecific competition can involve direct interference such as ‘scramble competition’ between buffalo ( *Syncerus caffer* ) and elephants ( *Loxodonta africana* ) arriving simultaneously at feeding patches, or competitive displacement, where elephant consumption was reduced at grazing sites previously visited by buffalo (De Boer and Prins 1990). Niche partitioning is also evident in the trophic dimension, where dietary overlap is influenced by rumen morphology and species‐specific plant selection (Redjadj et al. 2014). In African savanna systems, for example, large herbivores partition resources along a grazer‐browser continuum, differing in their reliance on grasses versus woody plants (Kartzinel et al. 2015). Similarly, in a recovering ecosystem in Mozambique, interspecific dietary overlap was greatest among grazers in homogeneous habitats, while niche separation was more pronounced among browsers in heterogeneous habitats, with abundant populations also showing greater individual dietary variation (Pansu et al. 2019). Taken together, these examples highlight how diet is a central component of partitioning, making the trophic dimension particularly important, as foraging provides energy and nutrients for body maintenance, thermoregulation, growth, and reproduction. The quality and quantity of available foods affect body condition (Couturier et al. 2009; Felton, Holmström, et al. 2020) and survival (Verdolin 2006) and have implications for fitness (Ripple et al. 2001) and population dynamics (White 1983).

Differences in diet between large herbivores are determined by several factors. These include herbivore distribution, vegetation heterogeneity (Cromsigt and Olff 2006; Van Wieren and Langevelde 2008), herbivore body mass (Bell 1971; Geist 1974; Jarman 1974), and in the case of ruminants, which include all deer species, their morphophysiological feeding type (see Hofmann 1989). The latter refers to a suite of suggested adaptations that allow for the utilization of different plant functional groups, placing ruminants along a continuum from concentrate selectors (often also referred to as ‘browsers’) to grass and roughage eaters (‘grazers’).

More recently, a refined concept of comparative ruminant digestive physiology has emerged, distinguishing between ‘Moose‐type’ or ‘Cattle‐type’ ruminants (Clauss et al. 2010). ‘Cattle‐type’ ruminants exhibit a pronounced separation in the retention time of fluids versus small particles in the reticulorumen and can occupy both the grazing and mixed feeding niches (Codron and Clauss 2010). This digestive strategy also confers a higher capacity to exploit human‐provided novel resources such as crops and pasture meadows, as illustrated by red deer in Norway (Unsgård et al. 2025). In contrast, ‘Moose‐type’ ruminants show little differentiation between fluid and particle digesta passage and are generally considered non‐grazers, restricted to browsing (Van Wieren 1996).

Two related metrics have become established tools among researchers for quantifying the utilization and partitioning of food resources: dietary niche width and dietary overlap. Dietary niche width refers to the range of utilized forage taxa and provides a measure for how generalized or specialized individuals or species are (Devictor et al. 2010). This has implications for how they respond to environmental changes and the extent to which sympatric species may compete (Bison et al. 2015). For example, species with a wide dietary niche width might be superior competitors that are able to persist in a wide range of habitats, whereas species with narrow niches are more vulnerable to extinction (Clavel et al. 2011). Dietary niche width is linked to diet overlap (as similar diets result in higher overlap), which, together with overlap in habitat use, is a prerequisite for competition under conditions of resource limitation (De Boer and Prins 1990; Putman 1996). Understanding what drives diet overlap is therefore central to the sustainable management of multi‐species communities. For example, in the link between foraging and population dynamics, Illius and O'Connor (2000) highlighted the critical role of ‘key resources’ on which large herbivores depend for survival. If different herbivores rely on the same plant species as key resources, it may heighten the risk for competition (Redjadj et al. 2014).

**FIGURE 1:**
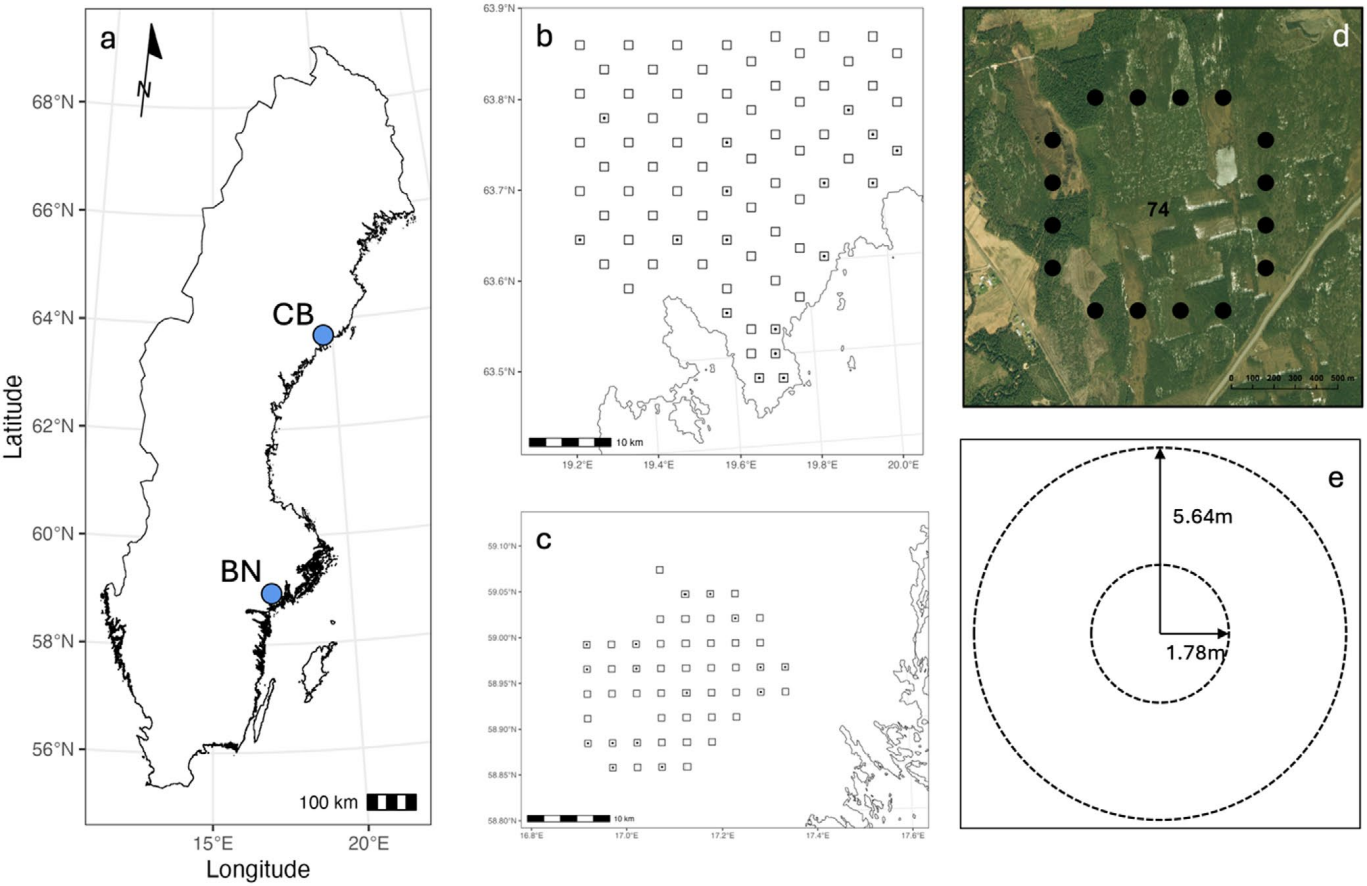
Location of the Coastal‐boreal (CB) and Boreo‐nemoral (BN) landscapes in Sweden (a). Each landscape contained a grid of 1 × 1 km square transects; 76 in the CB landscape (b) and 50 in the BN landscape (c). Most transects were sampled only during the spring pellet group counts (for deer density measurement), but a subset of 33 transects (indicated by a dot at the center) was sampled on a bi‐monthly basis. Each transect contained 16 evenly spaced sampling plots for pellet group counts (d). At each plot, pellet groups were counted on 100 m^2^ (*r* = 5.64 m) or 10 m^2^ (*r* = 1.78 m), depending on species and landscape (e). Fecal samples were collected for DNA metabarcoding along the whole length of the transects.

Among abiotic factors, seasonal changes strongly influence resource use and partitioning among large herbivores (Abraham et al. 2022). In northern latitudes, where winters are long and harsh, food can become extremely limited due to plant dormancy and snow cover. Such seasonal constraints are expected to directly affect dietary niche width, selectivity, and diet overlap (Porter et al. 2022), as studied here in boreal and boreo‐nemoral landscapes between 58° and 64° N (Figure 1).

For instance, intraspecific overlap may increase in winter as individuals converge on the few resources still available (e.g., evergreen browse) once deciduous leaves and forbs are absent and graminoids and dwarf shrubs become snow‐covered. Interspecific overlap may follow the same pattern or instead decline if species segregate by feeding type to minimize competition; for example, moose as browsers shifts to conifers, while intermediate feeders such as red deer and fallow deer exploit graminoid‐based supplementary feeds like hay or silage.

Historically, the systematic study of trophic resource use in multi‐species communities has been hampered by the difficulties of extracting diet profiles of high taxonomic resolution from large numbers of samples (Rayé et al. 2011). Consequently, such studies are rare (Spitzer et al. 2020). Today, molecular methods for diet analysis (Pompanon et al. 2012) provide rapid, standardized, and increasingly affordable data, allowing long‐standing questions on trophic resource use and partitioning to be revisited with larger sample sizes and dietary profiles resolved to high taxonomic detail (De Barba et al. 2014; Kartzinel et al. 2015; Mata et al. 2024; Pansu et al. 2019; Ratkiewicz et al. 2024; Sato 2025).

In this study, we used fecal DNA metabarcoding (Taberlet et al. 2018) to investigate patterns of trophic resource use and partitioning in multi‐species ungulate communities, consisting of moose, roe deer, red deer, and fallow deer in Sweden. This focus is particularly relevant as the three smaller deer species (roe, fallow, and red deer) have markedly increased in number and distribution across Europe in recent decades (Apollonio et al. 2010; Deinet et al. 2013). These increases are largely attributable to the factors outlined earlier in the introduction, such as reduced winter severity, enhanced legal protection, abundant food in human‐modified habitats, and the historical eradication of large predators. In northern Sweden, this has led to the formation of assemblages between moose, fallow deer, and red deer without historical analogue.

Moose and roe deer are typically viewed as browsers (sensu Hofmann 1989) with a ‘moose‐type’ rumen physiology (Clauss et al. 2010) and a diet primarily consisting of dicots such as tree leaves, twigs, and forbs, typically referred to as ‘browse’ (Shipley 1999). Red deer and fallow deer are often referred to as intermediate feeders (Azorit et al. 2012; Krojerová‐Prokešová et al. 2010; Spitzer et al. 2020) with a presumptive rumen physiology closer to the ‘Cattle‐type,’ which allows for a wider range of dietary items including graminoids. Fallow deer, in particular, have been described as one of the most grazer‐like deer species (Hofmann 1989; Obidziński et al. 2013). Body mass may also contribute to niche separation by shaping feeding selectivity and access to forage. While larger herbivores are often assumed to cope better with low‐quality diets and smaller ones to rely on selective feeding (Van Soest 1996), Clauss et al. (2013) argued that a digestive advantage of higher body mass is not supported by empirical evidence. Instead, explanatory models should shift from physiological to ecological scenarios such as linking forage quality with forage availability and body mass with feeding selectivity.

We expected diet richness and dietary niche width to be larger in intermediate feeders (red deer and fallow deer) compared to the browsers (moose and roe deer) (*Hypothesis 1*). This is because intermediate feeders can exploit a wider range of dietary items, including both browse and graminoids, whereas browsers are constrained by a more specialized foraging strategy and the relatively low diversity of trees and shrubs at northern latitudes. Similarly, we expected trophic resource partitioning between browsers and intermediate feeders to be driven primarily by the proportion of graminoids in the diet, with moose and roe deer avoiding graminoids because their rumens are poorly adapted to digest them (Clauss et al. 2010) (*Hypothesis 2*). Further, we expected intra‐ and interspecific diet overlap to vary across species and seasons and be affected by deer population density, habitat diversity, and the proportion of arable land (*Hypothesis 3*).

## 2 Methods

### 2.1 Study Area

The study area encompassed two Swedish landscapes (Figure 1): a northern landscape in the boreal forest near the coast (hereafter coastal‐boreal landscape), and a central landscape in the boreo‐nemoral region (hereafter boreo‐nemoral landscape). The latter represents a transitional ecological zone, also referred to as the southern boreal forest region, situated between the boreal coniferous (taiga) forests and the temperate deciduous (nemoral) forests.

The coastal‐boreal landscape is characterized by cold winters with daytime temperatures of −5 to −10°C and 5–6 months of snow cover, with shallower snow depths along the coast than further inland. The boreo‐nemoral landscape has milder winters with daytime temperatures typically near 0°C and only 1–2 months of snow cover (Cromsigt et al. 2023). Seasons were defined to match major vegetation changes in our study area: winter (November–March) corresponded to frequent snowfall and plant dormancy, spring (April–May) to the period between snowmelt and leafing out, and the remaining months to the growing season, here grouped as ‘summer‐autumn’ because monthly fecal sample numbers were too low for finer separation.

Both landscapes are characterized by a mixture of forests, mires, and agricultural land. Common tree species in both landscapes include Scots pine, Norway spruce ( *Picea abies* ), birches (*Betula* spp.), poplars (*Populus* spp.), and willows (*Salix* spp.). The boreo‐nemoral landscape further includes oak (*Quercus* spp.) and European beech ( *Fagus sylvatica* ). The forest field layer is dominated by ericaceous shrubs (mainly of the genera *Vaccinium*, *Calluna*, and *Empetrum*). Agriculture is more extensive in the boreo‐nemoral landscape, consisting of small‐ to medium‐sized pastoral and arable farms with leys (temporary grasslands sown with grasses or legumes for fodder) as the main crop. Supplementary feeding, i.e., the deliberate provision of feed such as hay, silage, or root vegetables to wildlife by humans (Felton et al. 2022), is also more common here than in the coastal‐boreal landscape. As such, the boreo‐nemoral landscape offers a wider range of food resources, including agricultural crops and supplementary feed, and may also be subject to stronger seasonal fluctuations due to crop harvesting.

Moose, roe deer, red deer, and fallow deer occur sympatrically at both landscapes. Wild boar ( *Sus scrofa* ) currently occurs only in the boreo‐nemoral landscape, and reindeer ( *Rangifer tarandus* ) visit the coastal‐boreal landscape during the winter.

In both landscapes, we used previously established sampling grids (FOMA, ‘Fortlöpande miljöanalys’; Edenius 2012 and the Beyond Moose research program; Cromsigt et al. 2023) of 1 × 1 km square transects (76 in the coastal‐boreal landscape and 50 in the boreo‐nemoral landscape) spaced on average 3–6 km apart for annual pellet group counts. Each side of a square transect contained 4 sampling plots (total = 16).

### 2.2 Sample Collection

Fecal samples for DNA metabarcoding analysis were collected from 2015 to 2017 during the annual spring pellet group counts just after snowmelt (March–April in the boreo‐nemoral landscape and April–June in the coastal‐boreal landscape). One moose fecal pellet or 2–3 pellets from the smaller deer (corresponding to approximately equal volumes and 2–5 g of fresh fecal matter in total per sample) were taken from pellet piles and placed into sterile, airtight 20 mL scintillation tubes filled with silica gel desiccant (~1–3 mm, with indicator [orange gel], Merck KGaA, Germany) (Taberlet et al. 2018). We considered samples to be fresh if the pellets still had a wet, shiny surface and showed no signs of infestation by coprophages (Hemami and Dolman 2005). To prevent cross‐contamination between samples, we used disposable plastic spoons or nudged fecal pellets directly into the tubes, avoiding all contact with the collector and other samples. To minimize the possible effects of environmental contamination (e.g., pollen grains; Sato 2025), we selected fecal pellets from the middle of a pellet pile, i.e., from those that had been most shielded from the environment. We then stored the silica‐dried samples in the dark at room temperature until further processing.

To capture the seasonal variation in diets, we also collected samples on a subset of 33 transects (16 in the boreo‐nemoral landscape and 17 in the coastal‐boreal landscape; Figure 1) following a bi‐monthly scheme (i.e., alternately sampling half of the 33 transects each month) from September 2016 to November 2017.

Fresh samples were collected for moose, red deer, fallow deer, and roe deer along the whole length of each transect (4 km). We aimed at collecting 5 samples for each deer species per transect and visit. Because fecal pellets of similarly sized cervids can be difficult to distinguish in the field (Spitzer et al. 2019), species identification was putative and subsequently verified by metabarcoding for each sample. To avoid pseudoreplication and reduce the likelihood of repeatedly sampling the same individual, we placed at least 200 m between samples from the same putative species.

### 2.3 Deer Density Measurement

Pellet groups were counted on 16 evenly spaced, circular sampling plots (*r* = 5.64 m [100 m^2^, putative moose and red deer pellets], and *r* = 1.78 [10 m^2^, putative roe deer and fallow deer pellets], same center point; Figure 1e) on all transects during spring each year. From 2016 onward, all pellet groups were counted on 100 m^2^ plots in the coastal‐boreal landscape due to lower average densities of all species in that landscape. The center of a pellet group had to fall within the plot boundaries for it to be included in the count. For putative moose and red deer, a pellet group had to consist of ≥ 20 individual pellets, and of ≥ 10 pellets for putative roe deer and fallow deer. As we were mostly interested in the effect of overall deer density on resource partitioning, and because pellet groups from the smaller deer species cannot be reliably distinguished in the field (Spitzer et al. 2019), we combined the pellet group counts from all four deer species into a ‘cervid index’ (Felton et al. 2022) standardized to a unit of pellet groups / 100 m^2^. We only included transects of which at least 12 of the 16 plots (= 75%) had been surveyed in the analyses and removed two outliers (> 25 pellet groups / 100 m^2^; > 3 SD above the mean).

### 2.4 Fecal DNA Metabarcoding for Species Identification and Diet Composition

We applied DNA metabarcoding rather than traditional approaches such as microhistology, as it allows simultaneous identification of deer species and diet composition from the same fecal sample. This is important because without DNA confirmation, fecal pellets from similarly sized deer species can easily be misclassified (Spitzer et al. 2019). In addition, DNA metabarcoding reduces dependence on observer expertise, is more efficient for processing large sample sizes, and enables direct comparisons across studies where the same markers and protocols are used.

As with other technological advances, DNA metabarcoding has its limitations. These include marker constraints (Taberlet et al. 2007) and PCR amplification biases (Nichols et al. 2018; Pawluczyk et al. 2015), which can influence read abundances and potentially over‐ or underestimate diet components. In herbivore diets, the quantity and quality of DNA in fecal samples may also be affected by differences in digestibility or variation in chloroplast numbers across plant species and tissues. Nevertheless, when standardized protocols are applied consistently across experiments and ecological gradients, any biases should remain comparable, meaning that relative differences in diet composition reflect genuine ecological patterns even if exact proportions of consumed and detected items differ.

Despite these caveats, diet quantification with DNA metabarcoding performs at least as reliably as alternative methods (Taberlet et al. 2018) and has been shown to produce results comparable to stable isotope analysis (Kartzinel et al. 2015) and macroscopy (Nichols et al. 2016). All fecal samples in this study were processed following the same DNA extraction and metabarcoding protocols described below.

#### 2.4.1 DNA Extraction

Approximately equal amounts of dried dung (about one moose pellet or ~2 g) from each sample were homogenized by crushing between folded‐over pieces of aluminum foil, transferred into 20 mL scintillation vials, and immersed in 70% ethanol. To ensure thorough mixing, vials were placed in an ultrasonic bath (Branson 2200) for 90 s. From the resulting suspension, 1800 μL was pipetted into 2 mL microtubes and centrifuged for 10 min at 13,200 rpm (16,168 × *g*) to form a pellet. After removing the ethanol supernatant, 20 μL of Proteinase K and 180 μL of ATL buffer were added to the pellet. Samples were incubated for 30 min at 56°C, shaken every 10 min, and centrifuged again for 30 s at 3000 rpm (835 × *g*). DNA was then purified on a QIASymphony SP instrument using the DSP DNA Mini Kit (Qiagen, Hilden, Germany), following the manufacturer's protocol, with an elution volume of 100 μL.

#### 2.4.2 DNA Metabarcoding Markers and PCR Amplification

To ensure reliable amplification during PCR, metabarcoding markers must be short enough to account for DNA degradation typically found in fecal or other eDNA samples (Valentini et al. 2009). In this study, we targeted two taxonomic groups: mammals (to confirm fecal sample identity) and plants (to characterize diet composition). Mammalian DNA was amplified with the primer pair Mamm02_F and Mamm02_R (Giguet‐Covex et al. 2014; Taberlet et al. 2018), which amplifies a 60–84 bp fragment of the mitochondrial 16S gene. To minimize amplification of potential contaminant human DNA, we included a blocking oligonucleotide (P007_Blk_Homo, ccaaccGAAATTTTTAATGCAGGTTTGGTAGTT‐C3).

Plant DNA was amplified using the highly conserved generalist primer pair Sper01_F and Sper01_R (Taberlet et al. 2018), which targets the P6‐loop of the chloroplast trnL intron of seed plants (Spermatophyta). This marker is widely used in herbivore diet studies (Bison et al. 2015; Churski et al. 2021; Kartzinel et al. 2015; Nichols et al. 2016; Pansu et al. 2019; Ratkiewicz et al. 2024). The Sper01 primers are particularly suitable for degraded material due to their short amplicon length (10–220 bp, mean: 48 bp), while still providing good coverage and taxonomic resolution for seed plants (Taberlet et al. 2018).

To assign sequence reads to the corresponding sample after high‐throughput sequencing, we used 36 tags of eight nucleotides with at least five differences between each of them (available at www.oup.co.uk/companion/taberlet), which were added to the 5′ end of each primer. In total, 36 reverse and 32 forward tagged primers enabled 1152 PCR products (three 384‐well plates) to be pooled in a single library. Tags were preceded by 2–4 random nucleotides (NN—NNNN) to improve cluster detection and base calling during sequencing (Taberlet et al. 2018).

PCRs were performed on 384‐well PCR plates in 20 μL volumes containing 2 μL of DNA extract. For Sper01, reactions contained 10 μL of AmpliTaq Gold 360 master mix (Applied Biosystems), 0.5 μM of each primer, and 0.16 μL (20 mg/mL) of bovine serum albumin (BSA, Roche Diagnostic). For Mamm02, the mixture was identical, except that each primer was used at 0.2 μM and a blocking oligonucleotide was added at 2 μM. Cycling conditions included polymerase activation at 95°C for 10 min, followed by 40 (Sper01) or 45 (Mamm02) cycles of 95°C for 30 s, 50°C for 30 s, and 72°C for 60 s, and a final 7‐min elongation at 72°C. We ran three technical PCR replicates per sample for Sper01 (diet) and one replicate for Mamm02 (species ID). Each PCR plate included extraction controls (no template at DNA extraction step, *N* = 8), primer/template blanks (*N* = 20), PCR negative controls (nuclease‐free water, *N* = 3), and PCR positive controls (DNA from species not expected in our field samples: mammals—brown bear *Ursus arctos* ; plants—Madagascar jasmine *Stephanotis floribunda*; *N* = 2). As additional verification, we included fecal DNA from known‐origin samples (Lycksele Zoo, Sweden; hunter‐collected from harvested animals) for all target ungulate species.

#### 2.4.3 DNA Purification, Pooling of PCR Products, and Sequencing

PCR products were purified using the MinElute PCR purification kit, checked via capillary electrophoresis (QIAxel; Qiagen GmbH), and pooled in equivolume mixes before sequencing. Sequencing libraries were prepared according to the Metafast protocol (https://www.fasteris.com/en‐us/NGS/DNA‐sequencing/Metabarcoding) and sequenced on an Illumina HiSeq 2500 platform using a paired‐end approach (2 × 125 bp).

#### 2.4.4 Bioinformatics Analysis for Filtering and Taxonomic Annotation of Sequences

Sequence data were processed with OBITOOLS (https://metabarcoding.org/obitools/; Boyer et al. 2016). Forward and reverse reads were aligned and merged into consensus sequences using *illuminapairend*; reads with low alignment scores (< 40) were discarded. With *ngsfilter*, we identified primers and tags and assigned reads to samples. Identical sequences were dereplicated with *obiuniq* while retaining sample origin. Additional filtering removed singletons, ambiguous sequences containing “N” (IUPAC code), and sequences longer than expected for the primer sets (> 100 bp for Mamm02; > 220 bp for Sper01).

For the taxonomic annotation of sequences, we built a reference library for local mammals and plants by extracting relevant gene regions from the European Nucleotide Archive (ENA), supplemented with an arcto‐boreal plant and bryophyte database (Soininen et al. 2015; Sønstebø et al. 2010; Willerslev et al. 2014). Further data cleaning and downstream analyses were conducted in R (R Core Team 2022).

For mammal identification, we compared the relative frequency of molecular operational taxonomic units (MOTUs), which corresponded exactly to sequences in the mammalian reference library, to the distribution of MOTUs not matching any reference sequence. MOTUs representing < 1% of reads within a PCR were removed, as were MOTUs < 40 bp (mostly bacterial artifacts) and PCRs with < 500 reads, indicating poor amplification. Mammal species identity was assigned according to the most abundant MOTU in a sample that exactly matched a reference sequence. If more than one mammal species was detected, we only retained samples in which the dominant MOTU was at least twice as abundant as the second most abundant MOTU.

For plant identification (diet), we analyzed three PCR replicates per fecal DNA extract. To assess consistency, we calculated distances of replicates from their barycenters based on their sequence composition (PCR distances) and compared them to distances between barycenters (sample distances). Under optimal amplification, PCR distances should be small (= zero under hypothetical perfect conditions with identical amplification across PCR replicates) compared to sample distances. We log‐transformed sample distances for normality and used the 5th percentile as a quality threshold, discarding outlier PCR replicates exceeding this distance. We further applied a graph‐partitioning approach to visualize PCRs and controls, discarding replicates clustering with controls or showing poor amplification (< 1000 reads). Sequences passing these steps were retained as MOTUs, with read counts averaged across the remaining two or three PCR replicates per sample. MOTUs with < 95% similarity to their closest match in the reference library were considered likely artifacts (e.g., sequencing errors or chimeras) and excluded. Read counts of closely related MOTUs assigned to the same taxonomic rank (e.g., genus *Vaccinium*) were collapsed (summed) into a single MOTU, which was subsequently treated as representing the corresponding taxon at that rank. The final dataset was stored in a PostgreSQL relational database (https://www.postgresql.org) to facilitate ecological analyses.

#### 2.4.5 Quantification of Diet Composition

Absolute read counts were converted into relative read abundances (RRA) by dividing the read count of each MOTU by the total number of reads in that sample, so that RRA represents the proportional contribution of each MOTU within a sample and confers equal statistical weight across samples (Willerslev et al. 2014). MOTUs that did not contribute at least 2.5% in any sample were excluded as sporadic occurrences (Bison et al. 2015). RRA is increasingly used to quantify diet composition in DNA metabarcoding studies (Churski et al. 2021; Craine et al. 2015; Deagle et al. 2019; Kowalczyk et al. 2019) because it allows for analysis at the level of individual samples (diets). RRA has also been shown to produce conclusions consistent with alternative approaches such as frequency of occurrence (FOO), which is based on presence/absence data (Kartzinel et al. 2015; Kowalczyk et al. 2019; Willerslev et al. 2014). However, FOO can exaggerate the apparent importance of rare items (Pansu et al. 2019; Sato 2025). For these reasons, all quantitative diet results in our study, including overall diet composition, proportions of specific food items, and references to consumption, are based on RRA. While RRA may not strictly equate to ingested biomass and can be biased when small sample sizes or outlier samples dominate the data (Sato 2025; Sato et al. 2019), our large sample size minimizes this concern.

Because the trnL‐P6 plant barcode varies in taxonomic resolution across plant families (Taberlet et al. 2007), sequences could sometimes only be assigned to genus level or higher. Unless otherwise noted, analyses were conducted at the finest available taxonomic resolution (Appendix Table A1). When necessary to match diet data with the taxonomic resolution of forage availability in the field (for selectivity calculations; Figure 6) or to produce diet summary graphs using common plant names (21 food categories; Figure 3) relevant to forest and wildlife management, we grouped the corresponding MOTUs into those categories.

### 2.5 Forage Availability and Selectivity

Forage availability was measured alongside the bi‐monthly collections of fecal samples on the subset of 33 transects. Using the step‐point method (Coulloudon et al. 1999; Evans and Love 1957) along the whole length of the transect, we recorded vegetation hits on a pole within the browsing height ranges of the four deer species (roe deer: 0–1.5 m, fallow deer: 0–1.8 m, red deer: 0–2.3 m, and moose: 0–3 m; Nichols et al. 2015). Such vegetation hits are analogous to potential bites by foraging deer and were converted to proportions for each height range, transect, and visit. To match the diet data for calculating selectivity, we averaged forage availability for each height range, transect, and season. Selectivity was then calculated as Jacob's index D (Jacobs 1974):

$$D=\left(r-p\right)/\left(r+p-2\mathrm{rp}\right)$$

where *r* represents the proportion of a food item in the diet and *p* its relative availability in the environment. The index ranges from −1 to 1, with negative values indicating smaller proportions of a food item in the diet than its relative availability in the environment (hereafter referred to as ‘avoidance’), positive values indicating ‘preference’ or ‘selection for’ a food item, and zero indicating ‘neutral’ selection.

### 2.6 Statistical Analyses

All analyses were conducted in R version 4.2.1 (R Core Team 2022) with a significance level for statistical tests of *α* = 0.05.

#### 2.6.1 Diet Richness and Dietary Niche Width (Hypothesis 1)

Diet richness (S) was calculated as the number of MOTUs detected in a fecal sample. Because the data did not meet the assumptions of normal distribution, we used Dunn tests, a non‐parametric post hoc method, with the Benjamini–Hochberg adjusted *p*‐values to test for differences in diet richness between deer species.

Dietary niche width (DNW) was calculated using Hill numbers, which provide a straightforward way to measure diversity in diet by accounting for both common and rare taxa (Alberdi and Gilbert 2019; Jost 2006). This approach is well suited to DNA metabarcoding data and has been successfully applied in similar contexts (e.g., Alberdi et al. 2020). Following Jost (2006), we defined DNW as the exponential of the Shannon index, i.e., DNW = exp.(H'). We calculated population‐level DNW for each landscape, deer species, and season based on the corresponding average diets across all fecal samples.

#### 2.6.2 Trophic Resource Partitioning and Selectivity (Hypothesis 2)

To investigate trophic resource partitioning, we calculated Bray–Curtis dissimilarities between fecal samples based on square‐root (Hellinger) transformed RRA of plant MOTUs in each sample (Ratkiewicz et al. 2024) and visualized the resulting patterns with a PCoA (Mata et al. 2024). We then tested for differences between pairwise combinations of deer species by applying the factor fitting procedure (function *pairwise.factorfit()* with 10,000 permutations) from the package *RVAideMemoire* (Herve 2023), with Bonferroni correction of *p*‐values. To identify which plant taxa (MOTUs) contributed most strongly to the observed separation, we used the function *envfit()* from the package *vegan* (Oksanen et al. 2017) with 10,000 permutations and projected the six taxa with the highest R^2^ values (i.e., strongest correlations with the first two ordination axes) onto the ordination.

#### 2.6.3 Drivers of Intra‐ and Interspecific Diet Overlap (Hypothesis 3)

We used package *EcoSimR* (Gotelli et al. 2015) to calculate Pianka's index (Pianka 1973) as a measure of diet overlap. This index, frequently applied to characterize diet overlap (Azorit et al. 2012; Lovari et al. 2014; Pansu et al. 2019; Putman 1996), ranges from 0 (no overlap) to 1 (identical diets). We calculated both intra‐ and interspecific diet overlap at the transect scale for each season.

To test the effect of explanatory variables on intra‐ and interspecific diet overlap, we used beta regression (Ferrari and Cribari‐Neto 2004) from package *betareg* (Cribari‐Neto and Zeileis 2010), which is appropriate for response variables constrained to the unit interval [0,1] (Churski et al. 2021; Pfeffer et al. 2021). Explanatory variables included deer density, habitat diversity, and the proportion of arable land. Habitat diversity and the proportion of arable land were extracted from the Swedish National Landcover database (Naturvårdsverket 2019) within a 1 km radius from the center of each square transect. Habitat diversity was expressed as the Shannon index across habitat types, while the proportion of arable land was calculated as the fraction of this land‐use type within each transect. Prior to analysis, we checked for collinearity among the predictor variables, and no pairwise correlation exceeded 0.3.

## 3 Results

In total, 2568 fecal samples (1080 moose, 329 roe deer, 666 red deer, and 493 fallow deer) passed the DNA quality filtering criteria and were included in the analyses. Deer density was higher in the boreo‐nemoral landscape ($\bar{x}$ = 4.88 pellet groups × 100 m^−2^ ± 2.17 SD) compared to the coastal‐boral landscape ($\bar{x}$ = 0.43 pellet groups × 100 m^−2^ ± 0.62 SD) as was the proportion of arable land ($\bar{x}$ = 15.7% ± 14.4 SD; coastal‐boreal landscape: $\bar{x}$ = 5.3% ± 7.9 SD) and, to a lesser extent, habitat diversity ($\bar{x}$ H' = 2.10 ± 0.19 SD; coastal‐boreal landscape: $\bar{x}$ H' = 1.95 ± 0.17 SD).

### 3.1 Description of Diets

**FIGURE 2:**
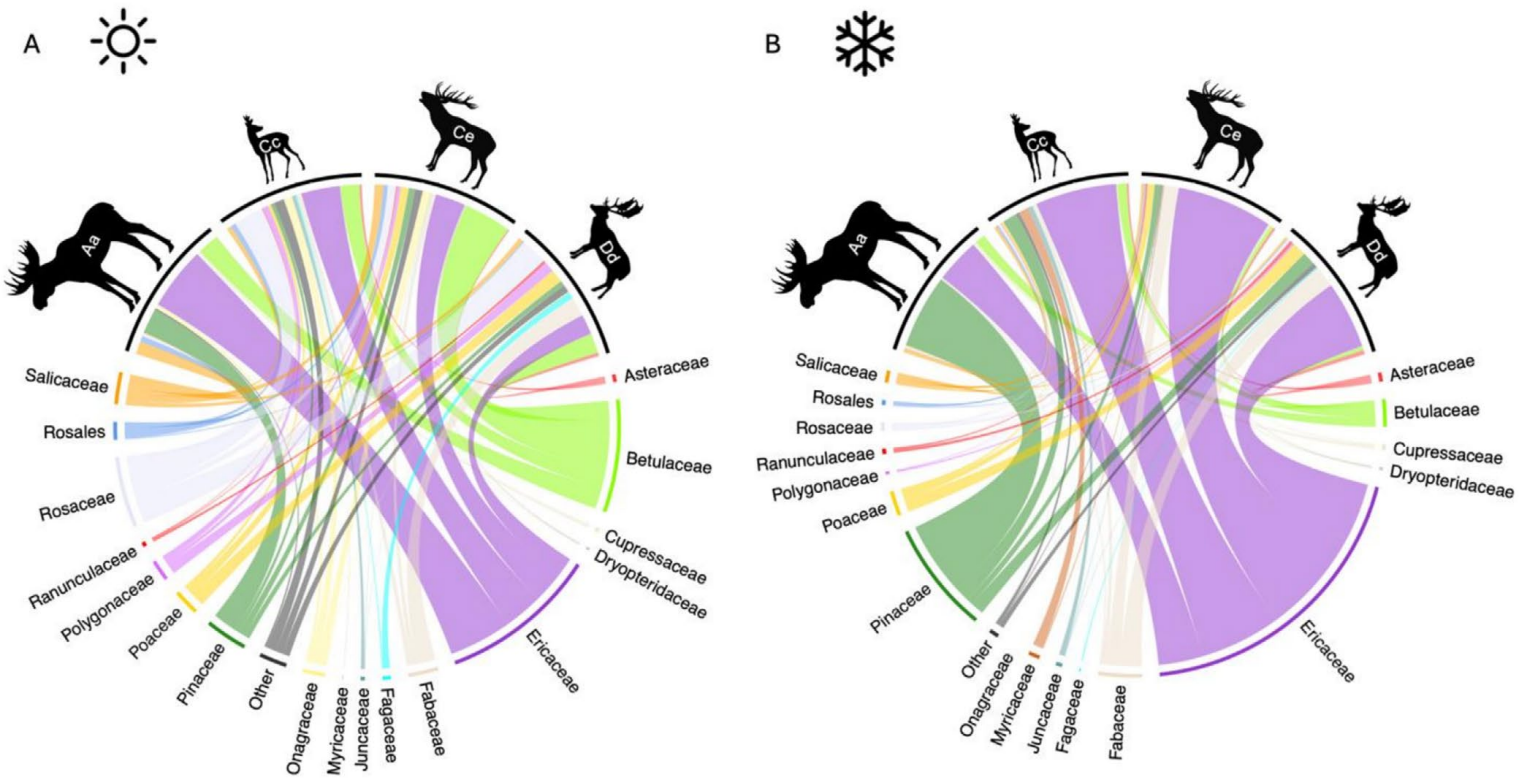
Chord diagrams showing the average diet composition for four deer species (clockwise, Aa = *Alces alces* [moose], Cc = *Capreolus capreolus* [roe deer], Ce = *Cervus elaphus* [red deer], and Dd = *Dama dama* [fallow deer]) during the growing season (spring to autumn, A) and the winter (B) at the taxonomic resolution of the plant family or higher. Only plant families which contributed > 1% to the average diet of at least one deer species are labeled, all others are summarized as ‘Other’. The colors below each silhouette represent the average diet for each species and link to the respective plant family. The length of the circle segment for each plant family represents the proportion at which the plant family is represented in the average diet of the ungulate community.

Based on the data from all 126 transects, across both landscapes, the diets across the four deer species comprised 210 MOTUs. At the level of plant family or higher taxonomic rank, those MOTUs corresponded to 77 categories (Appendix Table A1). Seventeen of those categories contributed at least 1% to the average diet (based on RRA) of at least one deer species (Figure 2). At the community level, deer diets were dominated by only a few plant families, primarily Ericaceae (corresponding largely to ericaceous shrubs of the genera *Vaccinium*, *Calluna*, and *Empetrum*), Pinaceae, Betulaceae, Rosaceae, Fabaceae, Poaceae, and Salicaceae both during winter and the other seasons.

**FIGURE 3:**
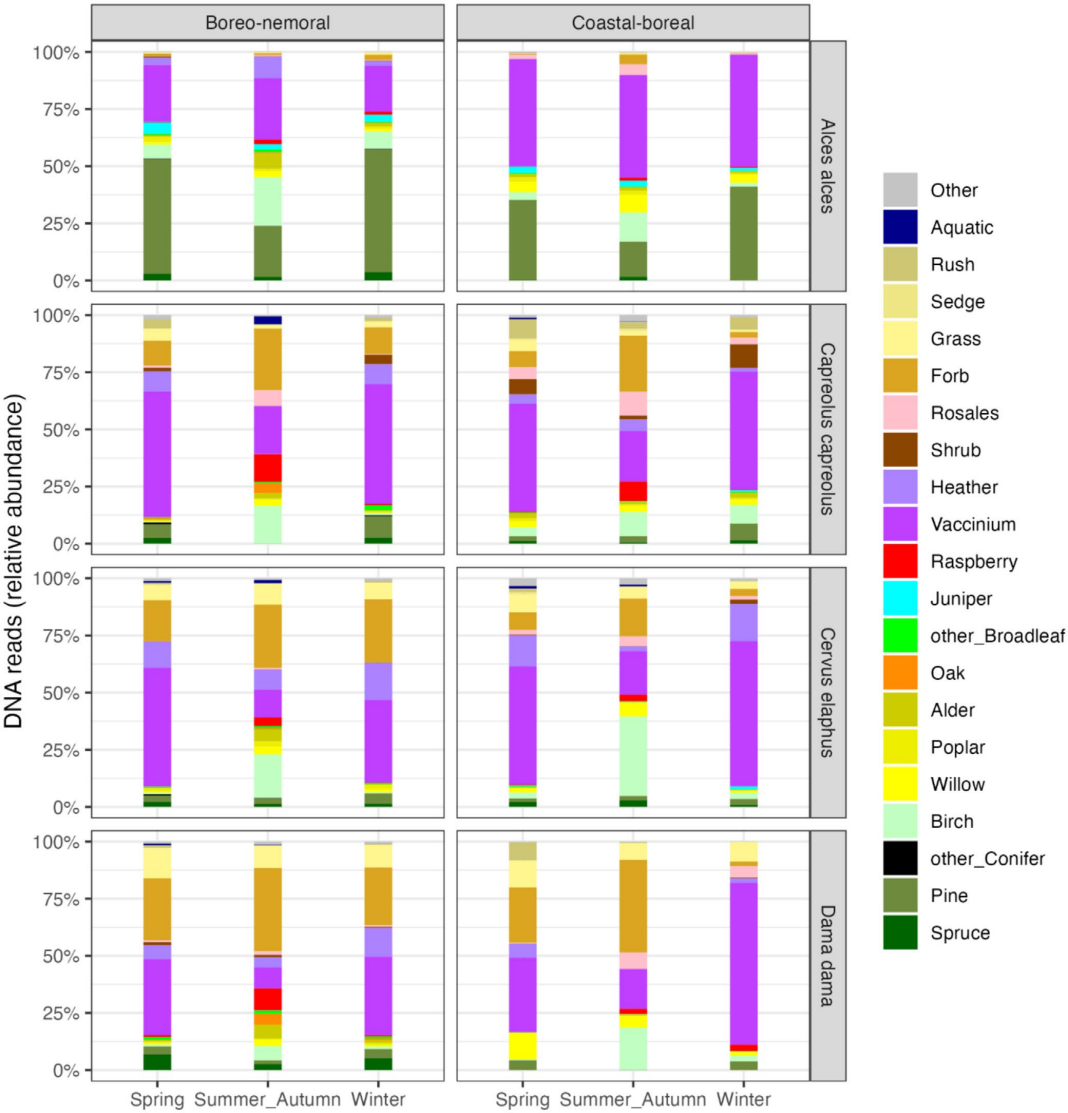
Average diet compositions (based on RRA) of four deer species during different seasons in the boreo‐nemoral and coastal‐boreal landscapes at the taxonomic resolution of 21 food categories.

The seasonal diet profiles at the resolution of 21 food categories (Figure 3) showed that among the ericaceous shrubs, *Vaccinium* spp. was consumed in high proportions throughout the year by all four deer species. During winter and spring, *Vaccinium* spp. frequently represented > 50% of DNA reads. *Vaccinium* spp. consumption during summer–autumn was generally lower but remained high for moose, particularly in the coastal–boreal landscape.

Moose diet during winter and spring was dominated by pine, especially in the boreo‐nemoral landscape, with some juniper, while both of these items were almost absent from the diet of the other deer species. Spruce was eaten in low amounts (typically < 5%) by all species, with fallow deer consuming the most. Spruce consumption in the boreo‐nemoral landscape was higher than in the coastal‐boreal landscape. Heather ( *Calluna vulgaris* ) frequently contributed 5%–10% to the diet of the smaller deer species, particularly red deer, but was less present in moose diets.

Birch and other broadleaf forage contributed to all deer diets throughout the year, with birch proportions being the highest during summer‐autumn, especially in red deer diets in the coastal‐boreal landscape ($\bar{x}$ = 35% ± 26 SD).

The amount of forbs in moose diets was generally low, reaching its highest value during summer‐autumn in the coastal‐boreal landscape ($\bar{x}$ = 4% ± 9 SD), but forbs contributed substantially to the diets of the smaller species, especially fallow deer, where approximately 40% of the average summer‐autumn diets were comprised of forbs in both landscapes. Notably, forbs also featured prominently in the winter diets of the smaller deer in the boreo‐nemoral landscape, contributing approximately 10% to roe deer diets and 25% to red deer and fallow deer diets.

**FIGURE A1:**
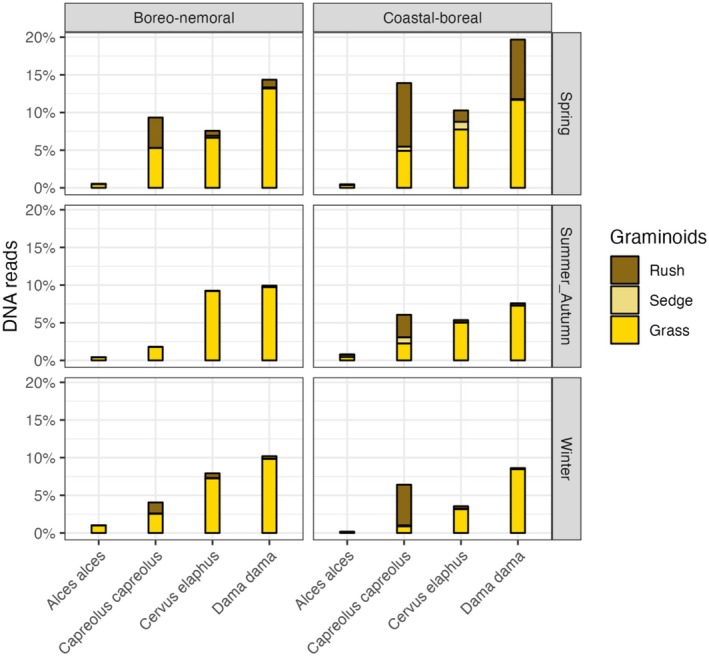
Relative abundances of graminoid DNA reads in the diet of four deer species during different seasons in the boreo‐nemoral and coastal‐boreal landscapes. Colors indicate the different types of graminoids, i.e., rushes (Juncaceae), sedges (Cyperaceae), and true grasses (Poaceae).

The proportions of graminoid DNA reads suggested generally low grass consumption. Graminoids were practically absent from moose diets and ranged from 5% to 20% in the diets of the smaller deer species. Graminoid utilization, although generally low, largely agreed with the commonly suggested gradient of ruminant feeding types (sensu Hofmann 1989) from ‘Moose‐type’ browsers (moose and roe deer) consuming no or very small amounts of graminoids toward increasing graminoid utilization by the more ‘Cattle‐type’ mixed feeders, red deer and fallow deer. Although graminoids contributed 10% and above to roe deer diets, especially during spring, the proportion of true grasses (Poaceae) was typically below 5% with roe deer instead largely feeding on rushes (Juncaceae) of the genera *Juncus* and *Luzula* (Appendix Figure A1).

### 3.2 Diet Richness and Dietary Niche Width (Hypothesis 1)

**FIGURE 4:**
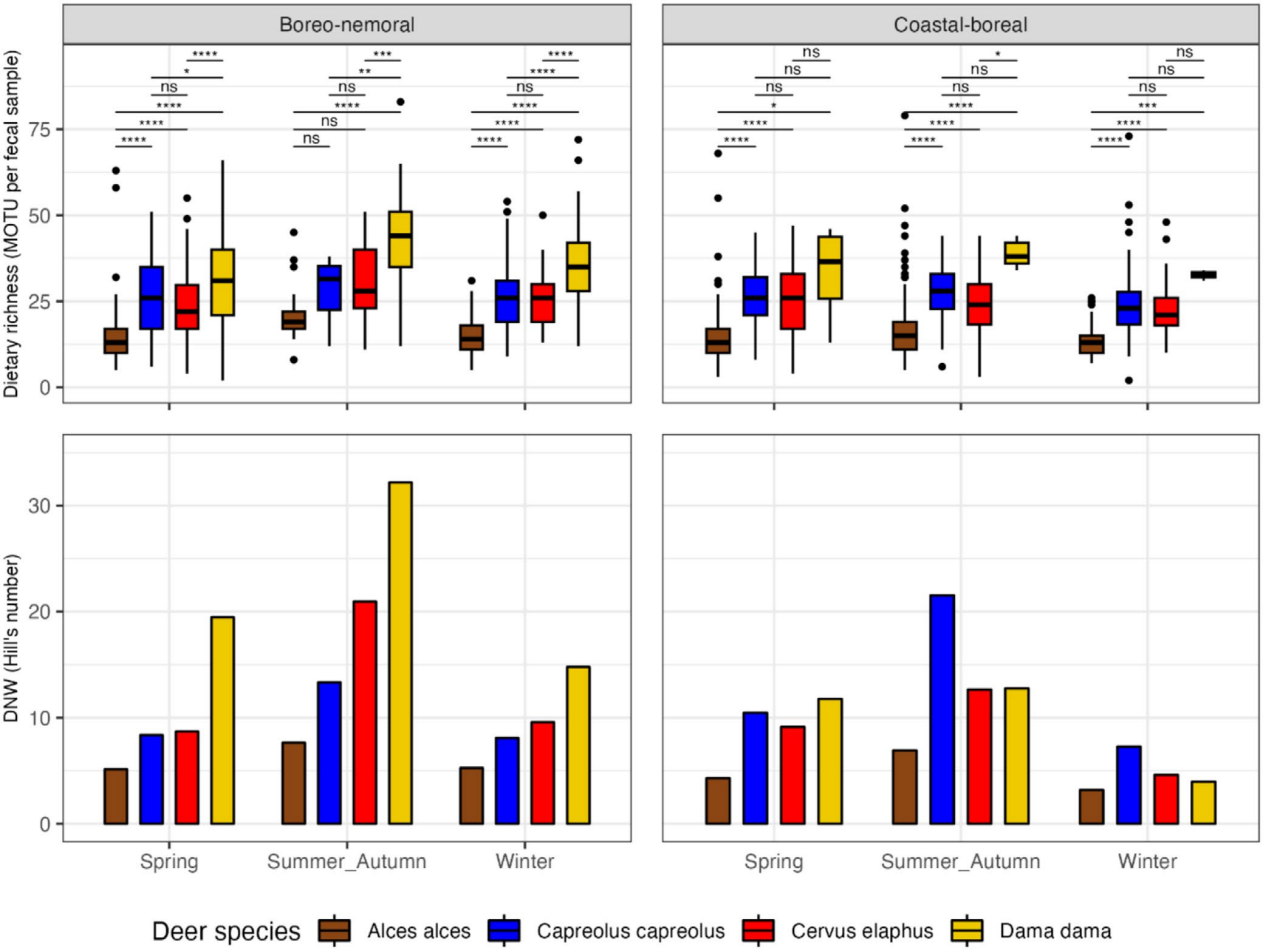
Dietary richness (top row) and dietary niche width (DNW, bottom row) of four deer species (indicated by colors) during different seasons in the boreo‐nemoral and coastal‐boreal landscapes. Differences in dietary richness among pairs of species are based on the Dunn test with the Benjamini–Hochberg correction of *p*‐values for multiple comparisons. Significant differences among species are indicated by asterisks (*p < 0.05, **p < 0.01, ***p < 0.001, ****p < 0.0001); “ns” denotes non‐significant differences (*p* > 0.05).

The variation in diet richness based on the number of MOTUs in individual fecal samples was similar within species and seasons across the two landscapes, while total dietary niche width was notably larger in fallow deer in the boreo‐nemoral landscape during all seasons and in red deer during summer–autumn and winter (Figure 4). Generally, diet richness was the lowest in moose (average number of MOTUs per sample 13–21), approximately equal in roe deer and red deer (25 to 28 MOTUs), and the highest in fallow deer (31 to 43 MOTUs). Similarly, dietary niche width consistently increased from moose, over roe deer and red deer to fallow deer in all seasons in the boreo‐nemoral landscape. In the coastal‐boreal landscape, the dietary niche width of moose was also always the lowest, while roe deer showed the broadest dietary niche width of all deer species during summer–autumn and winter (Figure 4).

### 3.3 Trophic Resource Partitioning and Selectivity (Hypothesis 2)

**FIGURE 5:**
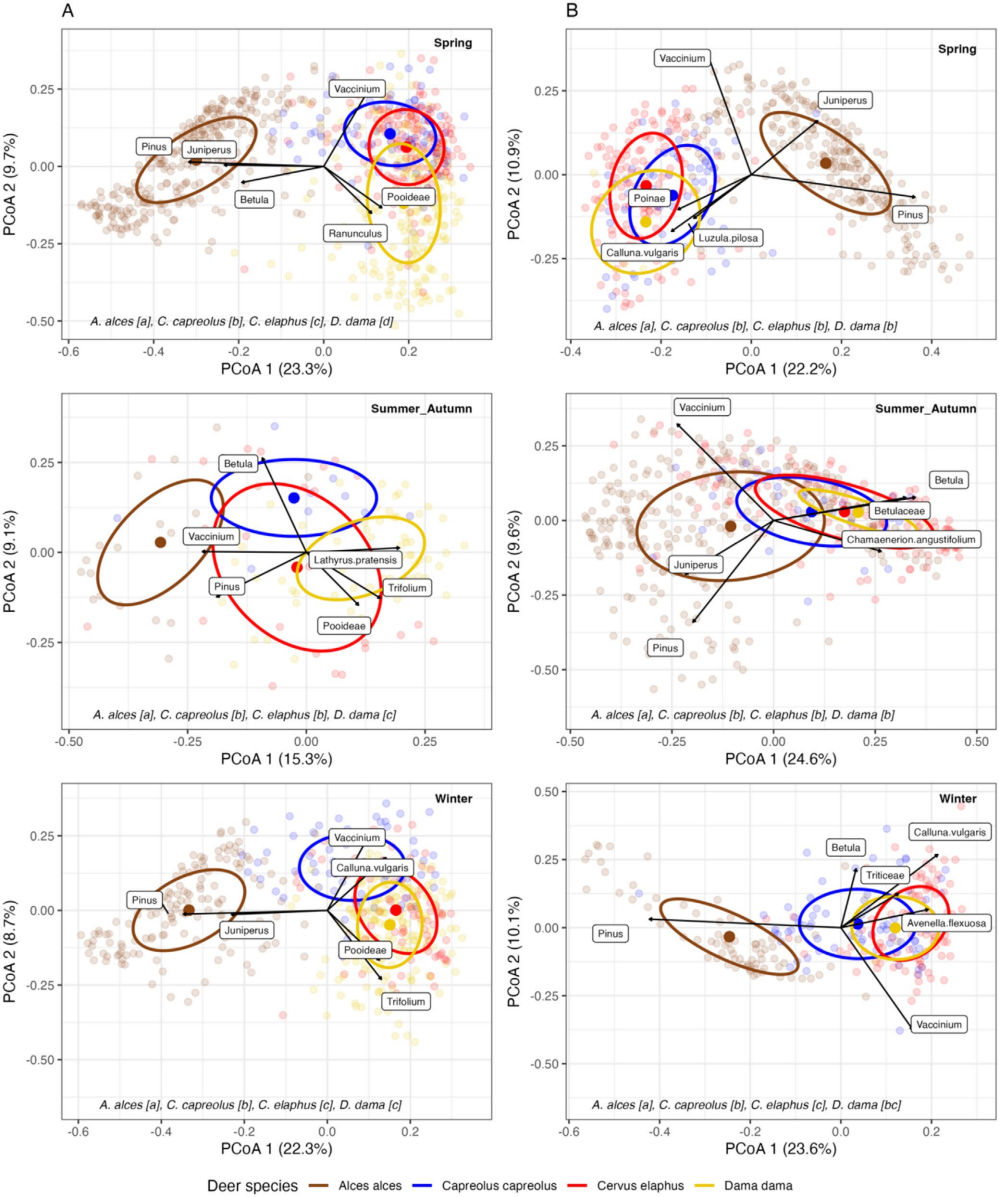
Principal coordinates analysis (PCoA) ordination of Bray‐Curtis dietary dissimilarities between deer diets during different seasons in the (A) boreo‐nemoral and (B) coastal‐boreal landscapes. Each small dot represents a fecal sample with colors indicating the deer species. Large dots show the group centroids for each species, and ellipses indicate one standard deviation around the centroids. Significant differences between species are denoted by different lowercase letters in square brackets. Black arrows show the direction of increase for the six plant taxa most strongly related to the first two ordination axes (scaled by *R* ^2^, higher *R* ^2^ values are represented by longer arrows).

PCoA generally showed clear separation of moose diets from the diets of the smaller deer species along the first axis, especially during spring and winter, driven by pine and juniper in moose diets (Figure 5). Although group centroids frequently differed significantly, the patterns of trophic resource partitioning (i.e., diet separation in the PCoA ordination space) between the smaller deer species were less clear.

During spring in the boreo‐nemoral landscape, the diets of roe deer and red deer were more associated with *Vaccinium* spp. than those of fallow deer, which were more associated with graminoids (Pooideae). In the coastal‐boreal landscape, the diets of the smaller deer were similar to each other and differed from moose mostly due to the presence of heather and graminoids in addition to the near absence of pine and juniper. Within moose, individual diets showed some separation along the second axis, corresponding to a gradient from pine to *Vaccinium* spp. in the diets.

In summer‐autumn, fallow deer diets in the boreo‐nemoral landscape differed from the other deer species due to the presence of graminoids and forbs such as meadow vetchling ( *Lathyrus pratensis* ) and clover (*Trifolium* spp.). The diets of roe deer and red deer were intermediate between moose and fallow deer and more associated with shrubs (*Vaccinium* spp.) and birch (*Betula* spp.) than fallow deer diets. In the coastal‐boreal landscape, graminoids were not a strong driver of diet separation during summer‐autumn; the diets of the smaller deer species were browse‐dominated and differed from moose diets mainly due to their higher birch and forb (e.g., fireweed *Chamaenerion angustifolium* ) content, less *Vaccinium* spp., and the near absence of pine.

The patterns of trophic resource partitioning in winter resembled those of spring in both landscapes, particularly with regard to the clear separation between the pine‐dominated diets of moose and those of the smaller deer species. Within the smaller deer, additional nuances were evident in winter diets. In the boreo‐nemoral landscape, roe deer diets differed from those of red deer and fallow deer through stronger associations with heather and *Vaccinium* spp., whereas red deer and fallow deer diets were more closely linked to graminoids and forbs (*Trifolium* spp.). In the coastal‐boreal landscape, the separation between roe deer and red deer diets was linked to higher contents of heather and the presence of cereal crops (Triticeae) in red deer diets compared to roe deer diets. The average fallow deer diet did not significantly differ from either roe deer or red deer, but showed greater overlap with red deer.

**FIGURE 6:**
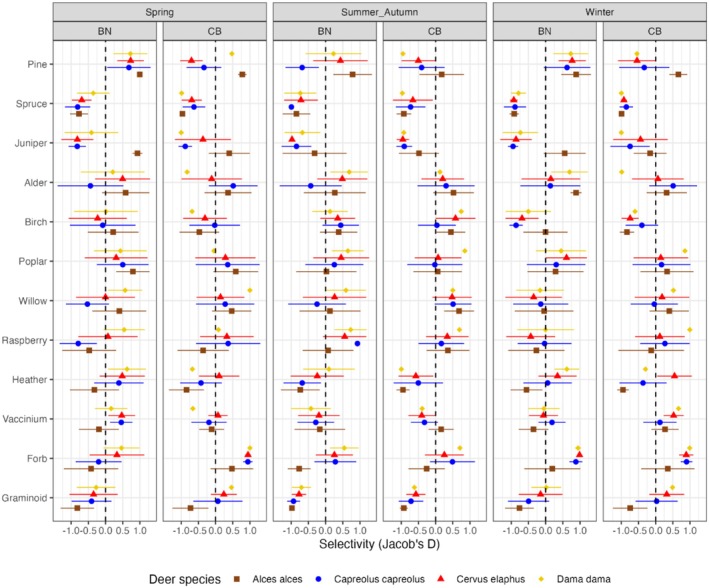
Selectivity (Jacob's D index) for 12 food categories by the four deer species (indicated by different colors and symbols) during different seasons in the two landscapes (BN = boreo‐nemoral landscape, CB = coastal‐boreal landscape) based on average diets per 1 × 1 km square transect. Shown are the mean values and standard deviation (error bars). Values without error bars correspond to instances where fecal samples were found on less than two of the transects where food availability was measured. Positive values of D indicate ‘preference,’ negative values ‘avoidance,’ and zero values ‘neutral selection.’ This figure provides a descriptive overview of selectivity; statistical significance of values is not shown due to uneven and occasionally small sample sizes, but values whose error bars do not overlap with zero can be regarded as especially strong indicators of preference or avoidance.

The analyses of selectivity showed that moose consistently consumed pine in higher proportions than would have been expected from the relative availability of pine in the environment in both landscapes during all seasons (i.e., showed ‘preference’), though less so in summer–autumn, especially in the coastal–boreal landscape (Figure 6). In the coastal–boreal landscape, the smaller deer species generally consumed less pine than would have been expected from its relative availability (i.e., showed ‘avoidance’) during all seasons (except for fallow deer in spring) but preferred pine in the boreo–nemoral landscape (except for roe deer in summer–autumn), albeit to a lesser degree than moose. Spruce was avoided by all deer species during all seasons in both landscapes. Juniper was generally avoided by the smaller deer species but preferred by moose in spring in both landscapes and during winter in the boreo–nemoral landscape.

Among the broadleaf trees, alder (*Alnus* spp.) tended to be eaten in proportion to its availability or was even moderately selected for except by roe deer in spring and summer–autumn in the boreo‐nemoral landscape. Selection for birch was mostly positive or neutral during summer–autumn for all deer species in both landscapes, with red deer and fallow deer showing notably high preference in the coastal‐boreal landscape. However, as the sample size for fallow deer in the coastal‐boreal landscape during summer–autumn was low (1 transect), the result for fallow deer should be interpreted with caution. During winter, birch was avoided by all deer species in both landscapes except moose in the boreo‐nemoral landscape, which consumed birch in proportion to its availability. Selection for poplars (*Populus* spp.) was generally positive or neutral across seasons and species. Similarly, willows (*Salix* spp.) were preferred by all deer species in the coastal‐boreal landscape except for roe deer, where selection was neutral in winter. In the boreo‐nemoral landscape, willows were avoided by roe deer during spring and summer–autumn but eaten in proportion to availability during winter. Moose preferred willows in spring and showed neutral selection during summer–autumn and winter.

For the ericaceous shrubs, heather was always avoided by moose but preferred by red deer during spring and winter in both landscapes. Similarly, fallow deer in the boreo‐nemoral landscape preferred heather during spring and winter and ate it in proportion to availability during summer–autumn. Selection for *Vaccinium* spp. was generally close to neutral. Notably, *Vaccinium* spp. was slightly preferred by all deer species during winter in the coastal‐boreal landscape but slightly avoided by moose in the boreo‐nemoral landscape, whereas the smaller deer species showed higher selectivity than moose for *Vaccinium* spp. during winter and spring in the boreo‐nemoral landscape. Conversely, selection for *Vaccinium* spp. was positive for moose and negative for the smaller deer species during summer–autumn in the coastal‐boreal landscape.

Forbs were always preferred by the smaller deer species, except for roe deer during spring in the boreo‐nemoral landscape. Graminoids were always avoided by moose and during summer–autumn also by the smaller deer species. Fallow deer showed the highest selectivity for graminoids among the four deer species. Selectivity for graminoids among the smaller deer species, especially red deer and fallow deer, was notably higher during spring and winter than during summer–autumn (Figure 6).

### 3.4 Drivers of Intra‐ and Interspecific Diet Overlap (Hypothesis 3)

Intraspecific overlap was generally highest in moose and lowest in fallow deer and decreased in summer–autumn in all deer species in both landscapes (Table 1).

**TABLE 1:** Average and standard deviation (SD) of the intraspecific dietary niche overlap (Pianka's index; 0 = no overlap, 1 = complete overlap) for four deer species during different seasons in the coastal‐boreal and boreo‐nemoral landscapes.

	*A. alces*	*C. capreolus*	*C. elaphus*	*D. dama*
Spring				
*Coastal‐boreal*	0.77 (0.17)	0.73 (0.21)	0.71 (0.27)	0.26 (n.a.)^+^
*Boreo‐nemoral*	0.74 (0.17)	0.74 (0.19)	0.65 (0.22)	0.55 (0.21)
Summer_Autumn				
*Coastal‐boreal*	0.64 (0.22)	0.44 (0.21)	0.58 (0.20)	0.38 (n.a.)^+^
*Boreo‐nemoral*	0.57 (0.36)	0.01 (0.005)^+^	0.41 (0.30)	0.30 (0.09)
Winter				
*Coastal‐boreal*	0.85 (0.13)	0.76 (0.21)	0.85 (0.12)	0.97 (n.a.)^+^
*Boreo‐nemoral*	0.81 (0.15)	0.76 (0.20)	0.65 (0.16)	0.57 (0.23)

*Note:* Values marked with a plus sign (+) are based on less than 4 replicates (transects) and should be interpreted with caution.

Interspecific overlap in the boreo‐nemoral landscape decreased in all species pairs during summer–autumn except between moose and red deer, where overlap increased in comparison to spring and winter (Table 2). During spring and winter, overlap was highest between red deer and roe deer and lowest between moose and fallow deer. Similarly, during summer–autumn, overlap was also lowest between moose and fallow deer but highest between red deer and moose.

**TABLE 2:** Average and standard deviation (SD) of the interspecific dietary niche overlap (Pianka's index; 0 = no overlap, 1 = complete overlap) for four deer species during different seasons.

	*A. alces*	*C. capreolus*	*C. elaphus*	*D. dama*
Spring				
*A. alces*	—	0.55 (0.29)	0.59 (0.24)	0.71 (0.21)^+^
*C. capreolus*	0.52 (0.24)	—	0.61 (0.30)	n.a.
*C. elaphus*	0.44 (0.23)	0.85 (0.14)	—	0.80 (0.08)^+^
*D. dama*	0.37 (0.18)	0.66 (0.24)	0.77 (0.21)	—
Summer_Autumn				
*A. alces*	—	0.61 (0.21)	0.56 (0.25)	0.70 (n.a.)^+^
*C. capreolus*	0.34 (0.33)	—	0.42 (0.27)	n.a.
*C. elaphus*	0.68 (0.30)	0.48 (0.35)	—	0.64 (n.a.)^+^
*D. dama*	0.26 (0.21)	0.39 (0.20)	0.35 (0.13)	—
Winter				
*A. alces*	—	0.61 (0.26)	0.61 (0.26)	0.78 (n.a.)^+^
*C. capreolus*	0.52 (0.21)	—	0.76 (0.28)	0.93 (n.a.)^+^
*C. elaphus*	0.44 (0.23)	0.77 (0.15)	—	0.98 (n.a.)^+^
*D. dama*	0.33 (0.20)	0.69 (0.23)	0.76 (0.23)	—

*Note:* For each season, the values for the coastal‐boreal landscape are shown in the upper triangular of the matrix and the values for the boreo‐nemoral landscape in the lower triangular and shaded in blue. Values with a plus sign (+) are based on less than 4 replicates (transects) and should be interpreted with caution.

In the coastal‐boreal landscape, the patterns of overlap were less consistent. A strong decrease in overlap during the summer–autumn was only observed for the species pairs red deer and roe deer, and red deer and fallow deer, with the latter being somewhat uncertain due to low sample sizes for fallow deer (Table 2). Overlap between moose and the smaller deer species showed less seasonal variation than in the boreo‐nemoral landscape and remained similar across the seasons. Overlap between moose and red deer, and moose and roe deer was also similarly high (~0.6, Table 2) across the seasons. Contrary to the boreo‐nemoral landscape, overlap between moose and fallow deer was higher than for moose and the other deer species, but this result should be treated with caution due to the small sample size for fallow deer in this landscape. Overlap was the lowest between red deer and roe deer during summer–autumn.

For the analyses of drivers of intra‐ and interspecific overlap, we report only the significant model coefficients (*β*, where negative coefficients indicate negative relation, and vice versa) and associated *p*‐values in the section below; the full results for all models are provided in the Appendix (Tables A2a and A2b).

In the coastal‐boreal landscape, beta regression of intraspecific overlap on the three explanatory variables (deer density, habitat diversity, and the proportion of arable land) showed significant relationships only for roe deer in spring (habitat diversity, *β* = −6.30, *p* < 0.01) and red deer in summer–autumn (proportion of arable land, *β* = −4.53, *p* = 0.01). Fallow deer was excluded from the analysis due to insufficient sample size.

In the boreo‐nemoral landscape, beta regression revealed significant relationships between intraspecific overlap and the explanatory variables for moose in summer–autumn (habitat diversity, *β* = 19.14, *p* = 0.01; proportion of arable land, *β* = −51.23, *p* < 0.01), red deer in winter (habitat diversity, *β* = −3.32, *p* = 0.02; proportion of arable land, *β* = 5.38, *p* = 0.01), and fallow deer during spring (proportion of arable land, *β* = −2.06, *p* = 0.04) and winter (deer density, *β* = 0.20, *p* < 0.01; proportion of arable land, *β* = −3.10, *p* < 0.01).

Interspecific overlap in the coastal‐boreal landscape was affected by the proportion of arable land (*β* = 9.64, *p* < 0.01) for the species pair moose–roe deer in spring, and for moose–red deer during summer–autumn by deer density (*β* = 0.56, *p* = 0.01), habitat diversity (*β* = 3.04, *p* = 0.03), and the proportion of arable land (*β* = −3.71, *p* = 0.046).

In the boreo‐nemoral landscape, we found relationships between interspecific overlap and the explanatory variables for moose–roe deer in winter (deer density, *β* = −0.19, *p* = 0.04), moose–fallow deer in spring (habitat diversity, *β* = 1.60, *p* = 0.02), roe deer–red deer in spring (deer density, *β* = 0.26, *p* = 0.02; habitat diversity, *β* = −3.78, *p* < 0.01; proportion of arable land, *β* = 3.57, *p* = 0.02) and winter (deer density, *β* = −0.30, *p* = 0.02), roe deer–fallow deer in winter (deer density, *β* = 0.32, *p* < 0.01; habitat diversity, *β* = −3.17, *p* = 0.01), and red deer–fallow deer during summer–autumn (deer density, *β* = −0.28, *p* < 0.01; proportion of arable land, *β* = 5.38, *p* < 0.01).

## 4 Discussion

Although we found a wide variety of food items spanning over 70 plant families at the cervid community level, diets were generally dominated by < 10 plant families. *Vaccinium* spp. shrubs contributed substantially to the diets of all four deer species during all seasons and in both landscapes. Birch and willow represented the most common deciduous tree browse in the diets during the growing season and for moose and roe deer also in winter. In contrast to the smaller deer, moose diets contained large amounts of pine during spring and winter (> 50% in the boreo‐nemoral landscape and 35%–40% in the coastal‐boreal landscape) and smaller amounts (~15%) during summer‐autumn. Forbs were used primarily by the smaller deer species during spring and summer‐autumn (ca. 10%–40%) and in the boreo‐nemoral landscape also during winter. The latter is likely due to less snow cover, larger proportions of arable land, and readily available supplementary foods such as hay and silage in the boreo‐nemoral landscape. The contribution of graminoids rarely exceeded 10% in the diets of the smaller deer species, and graminoids were practically absent from moose diets. One limitation to keep in mind, however, is that the Sper01 primers used in this study amplify only chloroplast DNA and thus do not capture non‐plant dietary items such as fungi and lichen, which are known to be consumed occasionally by the four deer species but typically represent a minor component compared to plants (Spitzer et al. 2020). An exception to this is reindeer, which, in contrast to most other ruminants, consume substantial amounts of lichens, especially in winter (Mathiesen et al. 2000; Storeheier et al. 2002), but were not included in this study as they occur only sporadically in parts of the northern landscape within our study area.

### 4.1 Diet Richness and Dietary Niche Width (Hypothesis 1)

We found partial support for the first part of Hypothesis 1 that diet richness on an individual level would be smaller in browsers (moose and roe deer) than in intermediate feeders (red deer and fallow deer). Moose feces consistently contained fewer MOTUs than those of the other deer species across all seasons and in both landscapes. Diet richness in roe deer, however, was consistently similar to that of red deer. Fallow deer feces contained the highest number of MOTUs on average (Figure 4).

The second part of our first hypothesis, i.e., that dietary niche width at the population level (DNW) would be larger in intermediate feeders than browsers, was supported in the boreo‐nemoral landscape, especially during summer–autumn, when the diversity of available foods in the environment is highest. In the coastal–boreal landscape, DNW of roe deer was similar to that of red deer and fallow deer in spring but exceeded DNW of both species during summer–autumn and winter. The difference to fallow deer could be explained by the smaller sample size of fallow deer feces in the coastal–boreal landscape (as fewer individual diet samples are likely to result in a narrower DNW) but not for red deer, where sample size was substantially higher than for roe deer (70 roe deer feces vs. 102 red deer feces during winter). Similarly, the consistently higher DNW of fallow deer in the boreo‐nemoral compared to the coastal–boreal landscape (Figure 4) is likely an artifact of the much lower sample size in the coastal–boreal landscape where fallow deer are less abundant and more spatially restricted, especially since diet richness in individual fallow deer fecal samples did not differ much between landscapes. In contrast, the higher DNW of red deer in the boreo‐nemoral landscape cannot be explained by sample size, as fewer red deer fecal samples were collected there than in the coastal–boreal landscape. Instead, the pattern is consistent with the niche variation hypothesis (Bison et al. 2015; Van Valen 1965), which proposes that wider DNW arises from greater inter‐individual dietary variation. Indeed, red deer in the boreo‐nemoral landscape showed an approximately 20% lower intraspecific diet overlap (suggesting larger inter‐individual variation) than in the coastal–boreal landscape (Table 1), indicating greater differentiation and thereby resulting in a higher DNW despite the smaller sample size. Moreover, the higher inter‐individual variation in red deer diets in the boreo‐nemoral landscape likely reflects their dietary plasticity as intermediate feeders, which makes them particularly adept at exploiting the more pronounced mosaic of fields and forests characteristic of this landscape compared to the coastal–boreal landscape.

### 4.2 Trophic Resource Partitioning and Selectivity (Hypothesis 2)

We also found some support for our second hypothesis that graminoids are an important contributor to dietary differences between browsers (moose and roe deer) and intermediate feeders (red deer and fallow deer). This was particularly evident in moose, whose diets consistently separated from the smaller deer due to the absence of graminoids and the presence of pine and juniper (Figure 5), reflecting their poor adaptation to digest grasses.

Diet of the smaller deer species tended to be similar; however, graminoids of the subfamily Pooideae separated fallow deer (typically regarded as one of the most grazer‐like deer species) from red deer and roe deer during spring in the boreo‐nemoral landscape. These graminoids were also more associated with red deer and fallow deer diets during summer–autumn and winter in that same landscape, further illustrating the capacity of intermediate feeders to exploit these resources. In the coastal‐boreal landscape, the diets of the three smaller deer species showed no significant separation in spring, but graminoids (Poinae and *Luzula pilosa* ) contributed to the significant separation from moose diets (Figure 5). Similarly, the diets of the smaller deer also did not significantly separate during summer–autumn, but separated from moose due to higher birch content in red deer diets, lower amounts of *Vaccinium* spp., and greater amounts of forbs such as fireweed. During winter, wavy hairgrass ( *Avenella flexuosa* ) contributed to the significant separation between browsing roe deer and intermediate‐feeding red deer diets, consistent with Hypothesis 2.

Selectivity for graminoids was generally also higher in the intermediate feeders red deer and fallow deer, whereas moose strongly avoided graminoids (Figure 6). Selectivity for graminoids was higher in roe deer than in moose, which may be owed to the fact that a substantial proportion of the graminoid fraction (> 50% in the coastal‐boreal landscape, Figure A1) in roe deer diets consisted of rushes such as wood‐rush ( *Luzula pilosa* ). These rushes may be more palatable to roe deer than true grasses (Poaceae). For example, wood‐rush has been shown to contain lower amounts of abrasive silica than grasses (Johnston et al. 1968).

We cannot fully rule out the possibility that the low proportion of graminoids we detected may stem from an unknown source of bias during DNA extraction or PCR amplification steps (Nichols et al. 2018) or the possible degradation of chloroplast DNA in supplementary feeds such as hay and silage. What opposes the latter is that we detected substantial amounts of forbs and graminoids in the winter diets of the smaller deer in the boreo‐nemoral landscape, where supplementary feeding is common, but not in the coastal‐boreal landscape, where it is rare.

Moreover, although the overall contribution of grasses to deer diets in our study area was small and all four deer species largely adopted a browser‐type diet rich in woody forage and forbs, the relative differences between them followed the classic gradient of ruminant feeding types (sensu Hofmann 1989): from browsers consuming little or no graminoids to increasing graminoid use by the intermediate feeders red deer and fallow deer. This pattern is consistent with observed diets of these species across Europe (Spitzer et al. 2020) and supports Van Wieren's (1996) hypothesis that ‘Moose‐type’ ruminants (Clauss et al. 2010) may be obligate non‐grazers, typically having less than 10% grass in their diets.

The general avoidance of spruce aligns well with other studies (Gill 1992; Hörnberg 2001; Månsson et al. 2007). However, the noticeable amounts of spruce in some deer diets, especially fallow deer, during spring and winter in the boreo‐nemoral landscape with high deer densities compared to its near absence of spruce in diets in the coastal‐boreal landscape with low deer densities (Figure 3), suggest that spruce may become a significant food source under conditions of resource competition. This has implications for forest management as spruce has increasingly replaced pine in plantations, partly motivated by an effort to minimize economic loss from browsing damage (Felton, Petersson, et al. 2020). Moreover, recent research indicates that spruce may also play a role in compensatory feeding by moose in the presence of sugary supplementary feeds such as beet roots (Felton et al. 2024).

### 4.3 Drivers of Intra‐ and Interspecific Diet Overlap (Hypothesis 3)

All three explanatory variables, deer density, habitat diversity, and the proportion of arable land significantly affected intra‐ and interspecific diet overlap consistent with Hypothesis 3, although the direction of effects differed between species and species pairs (Appendix Tables A2a and A2b).

At the intraspecific level in the coastal‐boreal landscape, diet overlap in roe deer declined with habitat diversity in spring, indicating that individuals exploited different food sources across habitats. Diet overlap in red deer was negatively related to arable land in summer–autumn, suggesting greater use of forbs and graminoids near fields compared to more forested areas. No effects were detected for moose, and the sample size for fallow deer was too small for analysis.

In the boreo‐nemoral landscape, intraspecific diet overlap in moose increased with habitat diversity but declined with arable land in summer and autumn, implying greater use of forbs, deciduous trees, and shrubs near fields, while the diversity effect may reflect that many land uses, such as artificial surfaces and roads, are unsuitable for foraging. Overlap in fallow deer diets in spring declined with arable land, likely due to field crops and supplementary feeding, but increased with deer density in winter, suggesting convergence on supplementary feed at high densities. Red deer overlap in winter increased with arable land but decreased with habitat diversity, perhaps indicating reliance on the same supplementary feed in agrarian areas.

At the interspecific level, we only found effects in the coastal‐boreal landscape for moose–roe deer and moose–red deer. Diet overlap between moose and roe deer increased with arable land in spring, suggesting shared use of emerging forbs and deciduous field‐edge browse. Moose–red deer overlap in summer–autumn increased with deer density and habitat diversity but declined with arable land, consistent with red deer being better able to exploit graminoids from fields. In this season, all deer species included substantial amounts of birch and *Vaccinium* spp. shrubs in their diets (Figure 3). The positive association between deer density and moose–red deer diet overlap is likely due to both using these abundant resources, suggesting that their current supply is adequate. Typically, one would expect the diet overlap between different feeding types to decline under food scarcity due to niche separation. This context‐dependent variation in overlap is consistent with the Competitive Exclusion Principle (Hardin 1960), which predicts that stable coexistence requires at least some degree of resource partitioning in space or time (e.g., Bøhn et al. 2008; Mishra et al. 2002).

In the boreo‐nemoral landscape in winter, moose–roe deer and roe deer–red deer overlap declined with deer density, likely reflecting differential use of supplementary feed (moose least able to utilize graminoid‐rich hay/silage, roe deer less able than red deer). In contrast, roe deer–fallow deer overlap increased with deer density, probably because both consumed more conifers (spruce and pine) than red deer.

In spring, roe deer–red deer overlap increased with deer density and arable land but declined with habitat diversity, suggesting different exploitation of foods in the various habitats. Moose–fallow deer overlap increased with habitat diversity, as moose diets became more similar to fallow deer in diverse habitats with less pine. In summer–autumn, red deer–fallow deer overlap declined with deer density, suggesting resource partitioning, but increased with arable land, indicating shared use of field resources.

Overall, no consistent patterns emerged, and although significant effects were detected, models generally explained less than half of the variation, with high *R* ^2^ values often coinciding with small sample sizes (e.g., roe deer in spring in the coastal‐boreal landscape, *R* ^2^ = 0.81, *N* = 10; Table A2a), requiring cautious interpretation.

This variability is in line with studies showing that dietary overlap among ungulates is strongly context‐ and season‐dependent. For example, in north‐eastern Poland, Ratkiewicz et al. (2024) found that moose, red deer, and roe deer partitioned diets during mild winters but converged on conifers in harsh winters, increasing potential competition. Mas‐Carrió et al. (2024) reported that European bison ( *Bison bonasus* ) and red deer exhibited higher niche overlap in areas of low plant diversity, while overlap decreased in more diverse, high‐quality habitats where species could diverge in diet choice. Our finding that arable land often reduced intraspecific overlap, although not consistently across all species and seasons, as red deer overlap increased with arable land in winter, is consistent with Abbas et al. (2011), who showed that roe deer in fragmented agricultural landscapes shifted diets toward cultivated crops that provided nutritional benefits but also led to differences among individuals in diet composition.

Habitat diversity effects were clearly species‐specific, with moose overlap increasing while roe deer and red deer overlap decreased. This resembles the species‐specific habitat responses described by Spitzer et al. (2020), who emphasized that although habitat type influenced intraspecific diet variation, forage‐type preferences remained stable and overlap varied primarily with season. Taken together, these studies support our conclusion that simple predictors rarely capture the complexity of trophic interactions. The same applies to deer density, which is often viewed as a major factor in deer impacts but may be too simplistic to serve as the main driver of trophic interactions in our study system. Forage availability, measured here only on a subset of transects as relative abundance, should be considered in future studies, ideally in biomass or energy terms.

Further knowledge of habitat‐specific diets is also needed. While fecal samples allowed associational analyses with habitat at the transect scale, diets could not be directly linked to feeding habitats. Future work should focus on habitat‐specific foraging choices (e.g., GPS‐tracked individuals, camera collars). Finally, species‐specific densities may be more informative than total deer density, but this is difficult to determine where dung from similar‐sized species cannot be reliably distinguished (Spitzer et al. 2019).

## 5 Conclusions

Our findings support the view of dietary plasticity among the four deer species, particularly the intermediate feeders, red deer, and fallow deer. Their substantial use of woody browse, especially ericaceous shrubs, highlights their adaptability to forest‐rich northern environments and may exert competitive pressure on food resources shared with roe deer and moose. Moose and roe deer appear less capable of exploiting graminoids available in agricultural areas or through supplementary feeding. This could potentially give red deer and fallow deer a competitive advantage in the evolving multi‐species deer communities in Sweden and other parts of northern Eurasia.

Our results also suggest that traditionally recommended measures to improve the food base for moose, such as retaining larger amounts of deciduous tree species during pre‐commercial thinning of young stands or planting willows, are likely to benefit the three smaller deer species as well. These measures should be maintained and intensified as deer populations continue transitioning into multi‐species communities. Our observed moose diets, which were distinct from those of smaller deer species due to their inclusion of pine, support previous findings that resource competition from smaller deer may drive moose to rely more on pine, or for longer periods (Spitzer et al. 2021). Conversely, abundant availability of pine may also reduce the competitive potential of smaller deer on moose.

The relatively high proportion of forbs and graminoids in the winter diets of smaller deer species in the boreo‐nemoral landscape suggests the utilization of supplementary feeding, which may be linked to the avoidance of resource competition between these species. This warrants further investigation. Additionally, we show that typically avoided spruce may become a more relevant food source under resource competition. Future research should explore these questions, particularly in the context of silviculture, supplementary feeding, deer density, and community composition—factors largely shaped by human management decisions. Therefore, our findings are valuable within a multi‐species adaptive management framework (Dressel et al. 2018), offering insights into managing deer populations in northern ecosystems.

## Author Contributions

**Robert Spitzer:** conceptualization (equal), data curation (lead), formal analysis (lead), investigation (equal), methodology (equal), visualization (lead), writing – original draft (lead), writing – review and editing (equal). **Eric Coissac:** conceptualization (supporting), data curation (equal), formal analysis (supporting), methodology (supporting), software (lead), writing – review and editing (equal). **Annika M. Felton:** conceptualization (equal), methodology (supporting), writing – review and editing (equal). **Marietjie Landman:** conceptualization (supporting), methodology (supporting), writing – review and editing (equal). **Navinder J. Singh:** conceptualization (equal), methodology (supporting), writing – review and editing (equal). **Pierre Taberlet:** conceptualization (supporting), data curation (supporting), investigation (equal), methodology (supporting), writing – review and editing (equal). **Fredrik Widemo:** conceptualization (equal), methodology (supporting), writing – review and editing (equal). **Joris P. G. M. Cromsigt:** conceptualization (equal), funding acquisition (lead), methodology (equal), project administration (lead), writing – review and editing (equal).

## Conflicts of Interest

The authors declare no conflicts of interest.

## Data Availability

The data that support the findings of this study are openly available on Dryad at DOI: https://doi.org/10.5061/dryad.0cfxpnwdd.

## Appendix A

**TABLE A1:** Molecular operational taxonomic units (MOTUs) identified by DNA metabarcoding of fecal samples from the four deer species (moose, roe deer, red deer, and fallow deer) in Sweden.

Plant family or higher	MOTU	Taxonomic rank
1. Aceraceae	*Acer*	Genus
2. Adoxaceae	*Sambucus*	Genus
3. Amaranthaceae	*Chenopodium*	Genus
	*Chenopodium suecicum*	Species
	*Oxybasis*	Genus
4. Amaryllidaceae	*Allium*	Genus
5. Apiaceae	*Aegopodium*	Genus
	Apioideae	Subfamily
	*Carum.carvi*	Species
	Oenantheae	Tribe
	Scandicinae	Subtribe
	Selineae	Tribe
6. Apocynaceae	*Vinca minor*	Species
	*Vincetoxicum hirundinaria*	Species
7. Araliaceae	*Hedera helix*	Species
8. Asteraceae	*Achillea millefolium*	Species
	Anthemideae	Tribe
	Asteraceae	Family
	Asterids	Clade
	Asteroideae	Subfamily
	Carduinae	Subtribe
	*Cirsium arvense*	Species
	Crepidinae	Subtribe
	Gnaphalieae	Tribe
	*Leontodon hispidus*	Species
	*Pilosella*	Genus
	*Scorzoneroides autumnalis*	Species
	Senecioninae	Subtribe
	*Tripleurospermum maritimum*	Species
9. Asterales	Asterales	Order
10. Aulacomniaceae	*Aulacomnium*	Genus
11. Betulaceae	*Alnus*	Genus
	*Alnus alnobetula*	Species
	*Betula*	Genus
	Betulaceae	Family
12. Boraginaceae	*Myosotis arvensis*	Species
13. Brachytheciaceae	Brachytheciaceae	Family
	*Sciuro hypnum*	Species
14. Brassicaceae	*Arabis alpina*	Species
	*Brassica oleracea*	Species
	Brassicaceae	Family
	*Braya*	Genus
	*Raphanus sativus*	Species
15. Bryaceae	*Bryum*	Genus
16. Cannabaceae	*Cannabis sativa*	Species
17. Caprifoliaceae	*Linnaea borealis*	Species
18. Caryophyllaceae	*Sagina*	Genus
	*Silene*	Genus
	*Spergula arvensis*	Species
	*Spergularia rubra*	Species
	*Stellaria*	Genus
	*Stellaria alsine*	Species
	*Stellaria pallida*	Species
19. Chenopodiaceae	*Beta vulgaris*	Species
20. Cistaceae	*Helianthemum nummularium*	Species
21. Cornaceae	Cornaceae	Family
	*Cornus suecica*	Species
22. Crassulaceae	*Hylotelephium telephium*	Species
	*Rhodiola rosea*	Species
	*Sedum album*	Species
	*Sedum sexangulare*	Species
23. Cucurbitaceae	*Bryonia dioica*	Species
24. Cupressaceae	*Juniperus*	Genus
25. Cyperaceae	*Carex*	Genus
	*Eriophorum*	Genus
	*Scirpus*	Genus
26. Dennstaedtiaceae	*Pteridium aquilinum*	Species
27. Dryopteridaceae	*Dryopteris*	Genus
28. Elaeagnaceae	*Hippophae rhamnoides*	Species
29. Ericaceae	*Andromeda polifolia*	Species
	*Arctostaphylos uva‐ursi*	Species
	*Calluna vulgaris*	Species
	*Chamaedaphne calyculata*	Species
	*Empetrum*	Genus
	*Orthilia secunda*	Species
	*Pyrola*	Genus
	*Pyrola rotundifolia*	Species
	*Rhododendron*	Genus
	*Vaccinium*	Genus
30. Euphorbiaceae	*Euphorbia palustris*	Species
31. Fabaceae	*Anthyllis vulneraria*	Species
	*Glycine max*	Species
	*Lathyrus*	Genus
	*Lathyrus pratensis*	Species
	*Lotus corniculatus*	Species
	*Lupinus*	Genus
	*Medicago*	Genus
	*Pisum sativum*	Species
	*Securigera varia*	Species
	*Trifolium*	Genus
	*Trifolium michelianum*	Species
	*Vicia*	Genus
	*Vicia faba*	Species
32. Fagaceae	*Fagus sylvatica*	Species
	*Quercus*	Genus
33. Geraniaceae	*Geranium*	Genus
	*Geranium robertianum*	Species
34. Grossulariaceae	*Ribes*	Genus
35. Hypericaceae	*Hypericum*	Genus
36. Hypnales	Hypnales	Order
37. Iridaceae	*Iris*	Genus
38. Juglandaceae	*Juglans regia*	Species
39. Juncaceae	*Juncus*	Genus
	*Juncus ranarius*	Species
	*Luzula*	Genus
	*Luzula pilosa*	Species
40. Lamiaceae	*Mentha*	Genus
	Mentheae	Tribe
41. Lamiales	Lamiales	Order
42. Linaceae	*Linum usitatissimum*	Species
43. Lycopodiaceae	Lycopodioideae	Subfamily
	*Lycopus europaeus*	Species
44. Lythraceae	*Lythrum salicaria*	Species
45. Menyanthaceae	*Menyanthes trifoliata*	Species
46. Mesangiospermae	Mesangiospermae	Clade
47. Myricaceae	*Myrica gale*	Species
48. Nymphaeaceae	Nymphaeaceae	Family
49. Oleaceae	*Ligustrum vulgare*	Species
	Oleeae	Tribe
50. Onagraceae	*Chamaenerion angustifolium*	Species
	*Epilobium*	Genus
51. Orobanchaceae	*Lathraea squamaria*	Species
	*Melampyrum pratense*	Species
	*Melampyrum sylvaticum*	Species
	*Rhinanthus*	Genus
52. Oxalidaceae	*Oxalis acetosella*	Species
53. Papaveraceae	*Corydalis solida*	Species
54. Pentapetalae	Pentapetalae	Clade
55. Pinaceae	*Abies*	Genus
	*Picea*	Genus
	Pinaceae	Family
	*Pinus*	Genus
	*Pinus contorta*	Species
56. Plantaginaceae	*Plantago*	Genus
	*Plantago lanceolata*	Species
	*Veronica chamaedrys*	Species
	*Veronica officinalis*	Species
	*Veronica serpyllifolia*	Species
57. Poaceae	Agrostidinae	Subtribe
	*Alopecurus geniculatus*	Species
	*Avena*	Genus
	*Avenella flexuosa*	Species
	Aveninae	Subtribe
	*Avenula pubescens*	Species
	*Dactylis glomerata*	Species
	*Glyceria*	Genus
	*Helictochloa hookeri*	Species
	*Holcus*	Genus
	*Hordeum*	Genus
	*Oryza sativa*	Species
	PACMAD clade	Clade
	*Phragmites australis*	Species
	*Piptatheropsis pungens*	Species
	*Poa*	Genus
	Poeae	Tribe
	Poinae	Subtribe
	Pooideae	Subfamily
	Stipeae	Tribe
	Triticeae	Tribe
58. Poales	Poales	Order
59. Polygonaceae	*Bistorta vivipara*	Species
	*Fallopia*	Genus
	*Persicaria*	Genus
	*Polygonum*	Genus
	*Rumex*	Genus
60. Polypodiaceae	*Polypodium vulgare*	Species
61. Primulaceae	*Hottonia palustris*	Species
	*Lysimachia*	Genus
	*Lysimachia thyrsiflora*	Species
	*Lysimachia vulgaris*	Species
	*Primula*	Genus
	*Trientalis*	Genus
62. Ranunculaceae	*Anemone*	Genus
	Anemoneae	Tribe
	*Ranunculus*	Genus
63. Rhamnaceae	*Frangula alnus*	Species
64. Rosaceae	*Agrimonia eupatoria*	Species
	*Alchemilla*	Genus
	*Comarum palustre*	Species
	*Filipendula ulmaria*	Species
	*Filipendula vulgaris*	Species
	Fragariinae	Subtribe
	*Geum*	Genus
	*Potentilla*	Genus
	*Prunus*	Genus
	*Pyrus communis*	Species
	*Rosa*	Genus
	Rosoideae	Subfamily
	*Rubus*	Genus
	*Sanguisorba officinalis*	Species
	*Spiraea*	Genus
65. Rosales	Rosales	Order
66. Rubiaceae	*Galium*	Genus
67. Salicaceae	*Populus*	Genus
	*Saliceae*	Tribe
	*Salix triandra*	Species
68. Saxifragaceae	*Heuchera richardsonii*	Species
	*Saxifraga*	Genus
	*Saxifraga granulata*	Species
69. Scrophulariaceae	*Limosella aquatica*	Species
70. Solanaceae	*Solanoideae*	Subfamily
71. Sphagnaceae	*Sphagnum russowii*	Species
72. Splachnaceae	*Splachnum vasculosum*	Species
	*Tetraplodon pallidus*	Species
73. Taxaceae	*Taxus baccata*	Species
74. Typhaceae	*Sparganium*	Genus
	*Typha*	Genus
75. Ulmaceae	*Ulmus*	Genus
76. Urticaceae	*Urtica*	Genus
77. Violaceae	*Viola*	Genus

*Note:* At the level of plant family or higher taxonomic rank, those MOTUs (*N* = 210) corresponded to 77 categories.

**TABLE A2a:** Estimates of model coefficients including standard errors (SE), *z*‐ and *p*‐values for predictor variables in beta regression with the intraspecific diet overlap in four deer species at two landscapes (CB = coastal‐boreal and BN = boreo‐nemoral landscape) as the response variable.

Response	Site	Season	*N*	Predictor	Estimate	SE	*z*	*p* (> |*z*|)	*R* ^2^_p_
Moose	CB	Spring	46	Deer density	−0.20	0.18	−1.11	0.27	0.03
				Habitat diversity	−0.31	0.81	−0.38	0.70	
				Arable land	−1.04	2.54	−0.41	0.68	
		Summer‐Autumn	50	Deer density	−0.04	0.19	−0.20	0.84	0.05
				Habitat diversity	−1.21	0.80	−1.52	0.13	
				Arable land	2.10	2.14	0.97	0.33	
		Winter	12	Deer density	0.10	0.21	0.45	0.65	0.22
				Habitat diversity	−0.50	1.63	−0.30	0.76	
				Arable land	−8.27	6.68	−1.24	0.22	
Roe deer	CB	Spring	10	Deer density	−0.001	0.16	−0.01	0.99	0.81
				Habitat diversity	−6.30	1.30	−4.83	**< 0.01**	
				Arable land	3.92	3.82	1.02	0.31	
		Summer‐Autumn	7	Deer density	−0.31	0.19	−1.58	0.11	0.35
				Habitat diversity	−0.37	1.81	−0.21	0.84	
				Arable land	1.06	4.65	0.23	0.82	
		Winter	9	Deer density	3.84	3.67	1.05	0.30	0.16
				Habitat diversity	−1.45	1.81	−0.80	0.42	
				Arable land	7.55	9.67	0.78	0.43	
Red deer	CB	Spring	25	Deer density	0.09	0.23	0.39	0.70	0.21
				Habitat diversity	2.29	1.24	1.84	0.07	
				Arable land	2.32	2.88	0.81	0.42	
		Summer‐Autumn	15	Deer density	−0.17	0.14	−1.17	0.24	0.48
				Habitat diversity	2.20	1.22	1.81	0.07	
				Arable land	−4.53	1.70	−2.66	**0.01**	
		Winter	9	Deer density	0.24	0.27	0.87	0.38	0.10
				Habitat diversity	1.85	2.40	0.77	0.44	
				Arable land	−12.50	8.84	−1.41	0.16	
Fallow deer	CB	—	—	—	—	—	—	—	—
Moose	BN	Spring	32	Deer density	−0.03	0.05	−0.65	0.52	0.05
				Habitat diversity	0.64	0.62	1.03	0.30	
				Arable land	0.14	1.21	0.12	0.91	
		Summer‐Autumn	5	Deer density	0.34	0.23	1.51	0.13	0.92
				Habitat diversity	19.14	7.83	2.45	**0.01**	
				Arable land	−51.23	16.24	−3.16	**< 0.01**	
		Winter	25	Deer density	−0.06	0.08	−0.73	0.47	0.04
				Habitat diversity	1.01	1.20	0.84	0.40	
				Arable land	−0.41	1.35	−0.30	0.76	
Roe deer	BN	Spring	16	Deer density	0.12	0.13	0.90	0.37	0.08
				Habitat diversity	−1.47	1.52	−0.97	0.33	
				Arable land	1.20	1.30	0.92	0.36	
		Summer‐Autumn	2	—	—	—	—	—	—
		Winter	12	Deer density	−0.02	0.16	−0.15	0.88	0.14
				Habitat diversity	−1.65	1.44	−1.14	0.25	
				Arable land	−0.97	1.78	−0.54	0.59	
Red deer	BN	Spring	24	Deer density	−0.08	0.09	−0.89	0.37	0.19
				Habitat diversity	−0.23	1.50	−0.16	0.88	
				Arable land	2.94	1.65	1.78	0.08	
		Summer‐Autumn	6	Deer density	0.57	0.33	1.76	0.08	0.37
				Habitat diversity	−2.76	4.02	−0.69	0.49	
				Arable land	−2.23	5.13	−0.43	0.66	
		Winter	14	Deer density	0.12	0.08	1.53	0.13	0.47
				Habitat diversity	−3.32	1.41	−2.36	**0.02**	
				Arable land	5.38	2.07	2.59	**0.01**	
Fallow deer	BN	Spring	34	Deer density	0.03	0.07	0.42	0.67	0.14
				Habitat diversity	0.61	0.72	0.84	0.40	
				Arable land	−2.06	0.98	−2.09	**0.04**	
		Summer‐Autumn	12	Deer density	−0.06	0.06	−0.94	0.35	0.07
				Habitat diversity	0.90	1.08	0.84	0.40	
				Arable land	−0.02	1.39	−0.02	0.99	
		Winter	29	Deer density	0.20	0.07	2.76	**< 0.01**	0.30
				Habitat diversity	−0.64	0.85	−0.75	0.45	
				Arable land	−3.10	1.05	−2.95	**< 0.01**	

*Note:* Model *R* ^2^ values are also provided. The sampling unit and number of replicates (*N*) correspond to 1 × 1 km square transects. Significant effects (*p* < 0.05) are marked in bold.

**TABLE A2b:** Estimates of model coefficients including standard errors (SE), *z*‐ and *p*‐values for predictor variables in beta regression with the interspecific diet overlap between pairs of four deer species at two landscapes (CB = coastal‐boreal and BN = boreo‐nemoral landscape) as the response variable.

Response	Site	Season	*N*	Predictor	Estimate	SE	*z*	*p* (> |*z*|)	*R* ^2^_p_
Aa—Cc	CB	Spring	17	Deer density	0.02	0.21	0.08	0.94	0.53
				Habitat diversity	−0.53	1.25	−0.42	0.67	
				Arable land	9.64	3.28	2.94	**< 0.01**	
		Summer‐Autumn	9	Deer density	−0.08	0.21	−0.38	0.70	0.25
				Habitat diversity	−2.85	1.82	−1.57	0.12	
				Arable land	3.21	5.76	0.56	0.58	
		Winter	12	Deer density	−0.07	0.24	−0.28	0.78	0.29
				Habitat diversity	−3.02	1.71	−1.79	0.07	
				Arable land	12.01	8.02	1.50	0.13	
Aa—Ce	CB	Spring	29	Deer density	−0.01	0.20	−0.04	0.97	0.16
				Habitat diversity	1.52	1.01	1.50	0.13	
				Arable land	2.54	2.54	1.00	0.32	
		Summer‐Autumn	21	Deer density	0.56	0.22	2.59	**0.01**	0.42
				Habitat diversity	3.04	1.38	2.21	**0.03**	
				Arable land	−3.71	1.87	−1.99	**0.046**	
		Winter	9	Deer density	0.11	0.31	0.35	0.73	0.05
				Habitat diversity	0.50	2.70	0.19	0.85	
				Arable land	3.16	10.11	0.31	0.76	
Aa—Dd	CB	—	—	—	—	—	—	—	—
Cc—Ce	CB	Spring	13	Deer density	0.31	0.25	1.24	0.21	0.29
				Habitat diversity	−0.16	1.61	−0.10	0.92	
				Arable land	4.22	3.52	1.20	0.23	
		Summer‐Autumn	8	Deer density	0.20	0.25	0.83	0.41	0.10
				Habitat diversity	0.22	2.10	0.11	0.92	
				Arable land	1.74	6.94	0.25	0.25	
		Winter	9	Deer density	0.35	0.32	1.11	0.27	0.36
				Habitat diversity	1.62	2.72	0.60	0.55	
				Arable land	3.65	10.22	0.36	0.72	
Cc—Dd	CB	—	—	—	—	—	—	—	—
Ce—Dd	CB	—	—	—	—	—	—	—	—
Aa—Cc	BN	Spring	23	Deer density	−0.01	0.10	−0.09	0.93	0.14
				Habitat diversity	−0.78	1.14	−0.69	0.49	
				Arable land	−1.73	1.18	−1.46	0.14	
		Summer‐Autumn	6	Deer density	0.89	0.49	1.81	0.07	0.35
				Habitat diversity	−17.15	9.80	−1.75	0.08	
				Arable land	−18.90	11.49	−1.65	0.10	
		Winter	18	Deer density	−0.19	0.09	−2.09	**0.04**	0.26
				Habitat diversity	0.54	1.03	0.52	0.60	
				Arable land	−1.14	1.10	−1.04	0.30	
Aa—Ce	BN	Spring	26	Deer density	−0.01	0.08	−0.18	0.86	0.04
				Habitat diversity	−0.94	0.95	−0.99	0.32	
				Arable land	0.61	1.43	0.43	0.67	
		Summer‐Autumn	6	Deer density	0.44	0.43	1.02	0.31	0.34
				Habitat diversity	−2.40	7.90	−0.30	0.76	
				Arable land	−0.63	17.34	−0.04	0.97	
		Winter	15	Deer density	−0.13	0.13	−1.01	0.31	
				Habitat diversity	−1.71	2.16	−0.79	0.43	
				Arable land	2.67	3.21	0.83	0.41	
Aa—Dd	BN	Spring	35	Deer density	−0.005	0.06	−0.08	0.94	0.15
				Habitat diversity	1.60	0.71	2.25	**0.02**	
				Arable land	−1.06	0.98	−1.08	0.28	
		Summer‐Autumn	12	Deer density	−0.01	0.13	−0.11	0.92	0.19
				Habitat diversity	−1.83	1.93	−0.95	0.34	
				Arable land	1.33	2.33	0.57	0.57	
		Winter	28	Deer density	0.03	0.07	0.47	0.64	0.05
				Habitat diversity	−0.97	0.83	−1.16	0.25	
				Arable land	−0.41	1.07	−0.38	0.70	
Cc—Ce	BN	Spring	18	Deer density	0.26	0.11	2.41	**0.02**	0.40
				Habitat diversity	−3.78	1.29	−2.92	**< 0.01**	
				Arable land	3.57	1.52	2.35	**0.02**	
		Summer‐Autumn	—	—	—	—	—	—	—
		Winter	11	Deer density	−0.30	0.13	−2.28	**0.02**	0.45
				Habitat diversity	2.43	1.40	1.74	0.08	
				Arable land	−2.86	2.21	−1.29	0.20	
Cc—Dd	BN	Spring	25	Deer density	0.08	0.11	0.69	0.49	0.07
				Habitat diversity	0.43	1.31	0.33	0.74	
				Arable land	−0.95	1.25	−0.76	0.45	
		Summer‐Autumn	8	Deer density	0.10	0.15	0.65	0.51	0.16
				Habitat diversity	−1.21	2.16	−0.56	0.58	
				Arable land	0.87	2.46	0.35	0.73	
		Winter	20	Deer density	0.32	0.11	2.93	**< 0.01**	0.32
				Habitat diversity	−3.17	1.14	−2.78	**0.01**	
				Arable land	−1.77	1.12	−1.57	0.12	
Ce—Dd	BN	Spring	24	Deer density	0.13	0.10	1.31	0.19	0.16
				Habitat diversity	0.70	1.11	0.63	0.53	
				Arable land	−2.17	1.63	−1.33	0.18	
		Summer‐Autumn	8	Deer density	−0.28	0.08	−3.67	**< 0.01**	0.83
				Habitat diversity	−1.17	0.88	−1.33	0.18	
				Arable land	5.38	1.27	4.24	**< 0.01**	
		Winter	15	Deer density	0.16	0.15	1.05	0.30	0.10
				Habitat diversity	−0.27	2.30	−0.12	0.91	
				Arable land	−3.05	3.47	−0.88	0.38	

*Note:* Model *R* ^2^ values are also provided. The sampling unit and number of replicates (*N*) correspond to 1 × 1 km square transects. Deer species are abbreviated as Aa (*Alces alces* moose), Cc (*Capreolus capreolus* roe deer), Ce (*Cervus elaphu*s red deer), and Dd (*Dama dama* fallow deer). Significant effects (*p* < 0.05) are marked in bold.
